# Weak-noise-induced transitions with inhibition and modulation of neural oscillations

**DOI:** 10.1007/s00422-018-0770-1

**Published:** 2018-07-11

**Authors:** Marius E. Yamakou, Jürgen Jost

**Affiliations:** 1 Max-Planck-Institut für Mathematik in den Naturwissenschaften, Inselstr. 22, 04103 Leipzig, Germany; 2 Fakultät für Mathematik und Informatik, Universität Leipzig, Augustusplatz 10, 04109 Leipzig, Germany; 3 Santa Fe Institute for the Sciences of Complexity, Santa Fe, NM 87501 USA

**Keywords:** Neuron model, Slow-fast dynamics, Bi-stability, Basin of attraction, Noise-induced

## Abstract

We analyze the effect of weak-noise-induced transitions on the dynamics of the FitzHugh–Nagumo neuron model in a bistable state consisting of a stable fixed point and a stable unforced limit cycle. Bifurcation and slow-fast analysis give conditions on the parameter space for the establishment of this bi-stability. In the parametric zone of bi-stability, weak-noise amplitudes may strongly inhibit the neuron’s spiking activity. Surprisingly, increasing the noise strength leads to a minimum in the spiking activity, after which the activity starts to increase monotonically with an increase in noise strength. We investigate this inhibition and modulation of neural oscillations by weak-noise amplitudes by looking at the variation of the mean number of spikes per unit time with the noise intensity. We show that this phenomenon always occurs when the initial conditions lie in the basin of attraction of the stable limit cycle. For initial conditions in the basin of attraction of the stable fixed point, the phenomenon, however, disappears, unless the timescale separation parameter of the model is bounded within some interval. We provide a theoretical explanation of this phenomenon in terms of the stochastic sensitivity functions of the attractors and their minimum Mahalanobis distances from the separatrix isolating the basins of attraction.

## Introduction

Fixed points, periodic, quasiperiodic or chaotic orbits are typical solutions of deterministic nonlinear dynamical systems. Multi-stability is common, as a dynamical system typically possesses two or more mutually exclusive stable solutions (attractors). For a given set of the parameters, coexistent stable states represented by different (or identical) types of attractors in the phase space of the system are by topological necessity separated by some unstable states. In neurodynamics for example, spiking neurons may possess coexistent quiescent (fixed point) and tonic spiking states (limit cycle) (Paydarfar et al. 2006), or distinct periodic and chaotic spiking states (Cymbalyuk and Shilnikov 2005). A given state can be reached if the system starts from a set of initial conditions within the state’s basin of attraction. Otherwise, an external perturbation can be used to switch the system from one stable attractor to another. When noise is introduced into the system, random trajectories can visit different stable states of the system by jumping over the unstable ones.

Important and challenging problems in multi-stable systems are to find the residence times of random trajectories in each stable state and its statistics, and the critical value of the noise amplitude and control parameters at which noise-induced jumping becomes significant. The analytical treatment of such problems based on the Fokker–Planck equation (FPE) becomes complicated for *n*-dimensional dynamical systems, $n\ge 2$, and therefore, various approximations were developed and are now commonly used (Van Kampen 2007; Freidlin and Wentzell 1998).

The quasi-potential method gives exponential asymptotics for the stationary probability density. In the vicinity of the deterministic attractor, the first approximation of the quasi-potential is a quadratic form (Mil’shtein and Ryashko 1995), leading to a Gaussian approximation of the stationary probability density of the FPE. The corresponding covariance matrix characterizes the stochastic sensitivity of the deterministic attractor: its eigenvalues and eigenvectors define the geometry of bundles of stochastic trajectories around the deterministic attractors. The Gaussian distribution centered on an attractor can be viewed as a confidence ellipsoid, while a minimal distance from this ellipsoid to the boundary separating the basins of attraction is proportional to the escape probability (Bashkirtseva et al. 2014). The appropriate measure for this distance is the so-called Mahalanobis distance (Mahalanobis 1936), the distance from a point to a distribution.

The residence time of random trajectories in a basin of attraction depends on two factors. The first factor is the geometry of the basin of attraction, e.g., the larger the distance is between an attractor and the separatrix isolating its basin of attraction, the longer is the residence time of phase trajectories in the basin. Second, the attractors are sensitive to random perturbations: the higher the stochastic sensitivity function (SSF) is, the higher is the probability to escape from the basin of attraction, and thus the shorter is the residence time (Freidlin and Wentzell 1998). Therefore, considering only the geometrical arrangement of stable attractors and the separatrix (the Euclidean distance between them) might not be sufficient for a theoretical explanation of a stochastic phenomenon, like the one to be investigated in the present work, and so the sensitivity of the attractors to random perturbations must also be taken into account. The Mahalanobis distance, which combines geometrical and stochastic sensitivity aspects of the dynamics, allows for a proper theoretical explanation of the transitions between attractors.

The effects of noise in neurobiological dynamical systems have been intensively investigated, for both single neurons and neural networks. Some of the most studied noise-induced phenomena are: stochastic resonance (SR) (Lindner et al. 2004; Longtin 1993; Collins et al. 1995), coherence resonance (CR) (Pikovsky and Kurths 1997), and noise-induced synchronization (Kim and Lim 2015). In SR, the neuron’s spiking activity becomes more closely correlated with a subthreshold periodic input current in the presence of an optimal level of noise. Pikovsky and Kurths (1997) showed that CR is basically SR in the absence of a periodic input current. In CR, noise can activate the neuron by producing a sequence of pulses which can achieve a maximal degree of coherence for an optimal noise amplitude if the system is in the neighborhood of its bifurcation value (Hopf bifurcation of a fixed point or saddle-node bifurcation on a limit cycle). We notice in these phenomena that noise has a facilitatory effect on the oscillations and leads only to increased responses.

More recently, it was discovered both experimentally (Paydarfar et al. 2006) and theoretically by (Gutkin et al. 2008, 2009; Tuckwell et al. 2009) (see also Tuckwell and Jost (2009)) that noise can also turn off repetitive neuronal activity. Gutkin et al. (2009) and Tuckwell et al. (2009) used the Hodgkin–Huxley equations in the bistable regime (fixed point and limit cycle) with a mean input current consisting of both a deterministic and random input component, to computationally confirm the inhibitory and modulation effects of Gaussian noise on the neuron’s spiking activity. They found that there is a tuning effect of noise that has the opposite character to SR and CR, which they termed inverse stochastic resonance (ISR). Very recently (August 2016), the first experimental confirmation of ISR and its plausible functions in local optimal information transfer was reported in (Buchin et al. 2016), where the Purkinje cells that play a central role in the cerebellum are used for the experiment.

During ISR, weak-noise amplitudes may strongly inhibit the spiking activity down to a minimum level (thereby decreasing the mean number of spikes to a minimum value), after which the activity starts and continuously increases with increasing noise amplitude (thereby monotonically increasing the mean number of spikes with increasing noise amplitude). In (Gutkin et al. 2009; Tuckwell et al. 2009), it is shown that *provided* the external deterministic input current (taken as bifurcation parameter) of the HH neuron model is below the Hopf bifurcation value of the fixed point and above the saddle-node bifurcation value of the limit cycles (i.e., in the parameter region of coexistence of these stable attractors), ISR occurs and persists in different situations: with random initial conditions (chosen from either the basin of attraction of the stable fixed point or the limit cycle), switching on the noise at a random time, and with a conductance-driven input.

In the present work, we will consider a stochastic slow-fast neuron model without an external deterministic input current component. Through bifurcation and slow-fast analysis, we will establish a bistable regime consisting of a stable fixed point and a stable limit cycle. We consider here a setting without an external deterministic input current component, because such an input would not make any critical difference in the qualitative behavior. What is critical is how the external and/or internal bifurcation parameters adjust to put the system in a bistable regime. Because of the slow-fast structure of the neuron model, we will analyze the effect of the parameter (the singular parameter) that induces different timescales in the model, on ISR. Essentially, ISR occurs when the noise strength matches the timescale separation, that is, when it can propel the dynamics at the right moment during the slow motion into the basin of attraction of the fixed point.

From numerical simulations, we observe that depending on the location of the initial conditions and on the value of the timescale separation parameter of the model equation, ISR could occur and be more pronounced for some values of the timescale separation parameter. More precisely, it is shown that ISR *always* occurs when the initial conditions are chosen from the basin of attraction of the stable limit cycle. When the initial conditions are in the basin of attraction of the stable fixed point, ISR disappears, except when the timescale separation parameter of the model lies within a certain interval. A theoretical explanation of this phenomenon is given in terms of the SSFs of the stable attractors and their minimum Mahalanobis distances from the separatrix.

This paper is organized as follows: In Sect. 2, we describe the origin of the slow and fact timescales in the FHN model, and mechanism underlying a spike generation. We describe up front the strategy we will use to investigate the weak-noise-induced phenomenon of ISR. In Sect. 3, we present the theoretical neuron model used to analyze ISR. In Sect. 4, we make explicit deterministic bifurcation analysis of the model equation and show how bi-stability consisting a stable fixed point and a stable limit cycle establishes itself. In Sect. 5, we make a stochastic sensitivity analysis of the stable attractors. In Sect. 6, we investigate ISR through numerical simulations and provide a theoretical explanation of the phenomenon using the results in Sect. 5. In Sect. 7, we have concluding remarks.

## The interaction of timescale separation and noise strength

In this paper, we investigate the mechanism behind ISR on the FHN neuron model. This model possesses two dependent variables and is a coarse-grained version of the most biophysically realistic, but also very complicated neuron model system, the HH system (Hodgkin and Huxley 1952) which has four dependent variables, the variable *V* for the membrane potential of the neuron, the potassium channel activation *n*, the sodium channel inactivation *h*, and the sodium channel activation *m*. The crucial point underlying FitzHugh (1961) is that these variables evolve on different timescales. *m* is very fast and can be equated to a constant, and the equation describing *m* then drops out. It has also been noticed experimentally that the sum of the potassium channel activation variable *n* and sodium channel inactivation variable *h* is almost constant during the action potential. Thus, the equations for *n* and *h* in the HH model can be fused into a single equation for a variable *w*. Thus, the HH model is reduced to a 2-dimensional model system governing the evolution of the membrane potential variable *v* and the ion channels variables *w*. Also, the qualitative behavior of *v* can be modeled by a cubic nonlinearity instead of the more complicated one in HH. Based on these observations, approximations, and the fact that the ion channels variable *w* evolves on a much slower timescale than the membrane potential variable *v*, a 2-dimensional slow-fast model (the FHN neuron model) with a slow variable (the recovery ionic current) and a fast variable (the membrane potential) is obtained,

1 $$\begin{aligned} \hbox {d}v=[v(a-v)(v-1)-w]\hbox {d}t,\,\, \hbox {d}w=\varepsilon (bv-cw)\hbox {d}t, \end{aligned}$$

here $\varepsilon$ is the timescale separation parameter.

Thus, the FHN model simplifies the HH model by projecting from the 4-dimensional phase space onto a plane with a weaker nonlinearity, but is still capable of reproducing the spiking dynamics of the HH neuron model. Mathematically, at the expense of numerical accuracy, the FHN neuron model allows for a rather complete mathematical understanding of the dynamical behavior and a geometrical explanation of important phenomena related to the excitability and spike generation mechanisms in neurons.

There is a powerful mathematical theory available, developed by Fenichel (Fenichel 1971, 1979), for analyzing multiple timescale (slow-fast) dynamical systems, and this theory fits FHN very well. It can explain many complex oscillatory phenomena such as spiking, bursting, and mixed-mode oscillations (Kuehn 2015; Bertram et al. 2010). By combining the slow-fast techniques with classical bifurcation analysis, one can geometrically understand the mechanism that underlies the spiking or non-spiking behavior of the FHN model in the absence and presence of noise. In this paper, we shall adopt this slow-fast analysis strategy to understand the ISR mechanism in the FHN model. Of course, since ISR is a stochastic phenomenon, the slow-fast scheme needs to be combined with tools from stochastic analysis, and we shall see how ISR emerges from the interaction of the timescale separation parameter $\varepsilon$ and the noise strength $\sigma$.

**Fig. 1:**
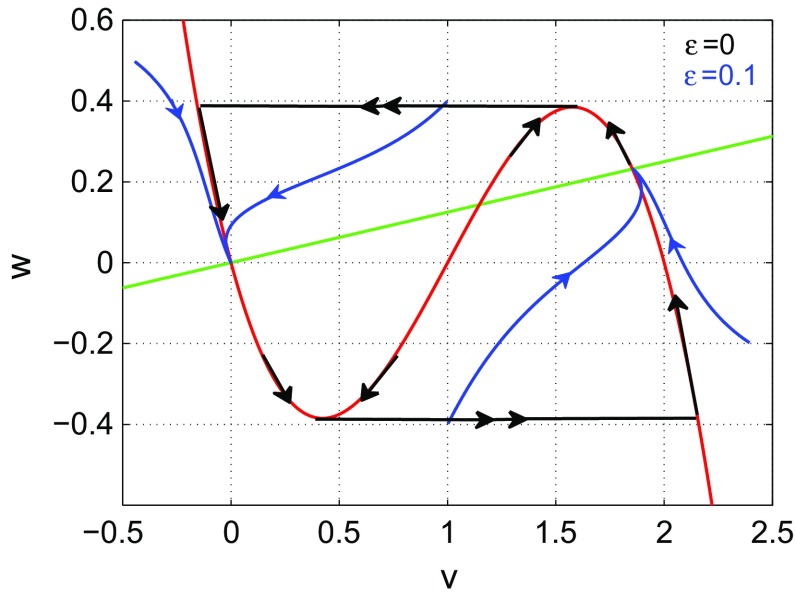
All the black trajectories (with single and double arrows) represent the solution of the slow flow on the critical manifold (the red curve) in the limit $\varepsilon =0$. These trajectories reach the fold points (at the extrema) of the critical manifold in finite forward time. At these fold points, trajectories jump forth and back on the left and right descending parts of the critical manifold, thereby generating a spiking behavior. The blue trajectories correspond to the flow (when $\varepsilon \ne 0$) on a perturbed manifold near the critical manifold according to Fenichel’s theorem. These trajectories converge to the stable fixed points $v_0 = 0$ and $v_2$ located at the intersection of the w- nullcline (the green line) and the decreasing parts of the critical manifold, without a limit cycle (spiking) occurring. Spiking occurs in the case $\varepsilon \ne 0$ when the there are no stable fixed points on the descending parts of the critical manifold as in Fig. 3b

The details of the corresponding slow-fast analysis are presented in mathematical “Appendix” of this paper, but here we want to describe the principle of the mechanism in words. When the timescale parameter $\varepsilon \rightarrow 0$, the fast *v*- and the slow *w*-dynamics become separated. The dynamics depends on the stability of the so-called critical manifold, the *v*-nullcline (see the red curve in Fig. 1), that is, whether it attracts or repels the fast *v*-dynamics. As long as it is stable, the slow *w*-dynamics then moves along this critical manifold (see the black almost vertical trajectories with single arrow in Fig. 1), but when it reaches a point where the slow manifold becomes unstable, a so-called fold point (extrema of the *v*-nullcline), it jumps away from it (see the black double arrow horizontal trajectories in Fig. 1) until it reaches another stable branch whence the process repeats. This is supposed to model the firing of the neuron. In the present case, since the equation for *v* has a cubic nonlinearity, the *v*-nullcline is the graph of a cubic polynomial with negative leading term. It has two descending parts which are stable and separated by an ascending, unstable part. We thus have two fold points, the local extrema of the cubic polynomial at which stability changes. Fenichel’s theorem then tells us that for small $\varepsilon$, the dynamics is a perturbation of that limiting dynamics (see the blue trajectories in Fig. 1).

The basic ingredient needed for ISR then is the coexistence of a stable fixed point and a stable limit cycle. In the slow-fast FHN model, the bi-stability regime needed for ISR is achieved when the fixed point (which exists for any value of the timescale parameter $\varepsilon$) sits on the unstable part of the critical manifold (the *v*-nullcline) and is itself stable for a suitable range of the timescale parameter $\varepsilon$. (As explained, the critical manifold represents the dynamical limit of the slow dynamics when $\varepsilon \rightarrow 0$, and unstability refers to that limiting situation.) This condition is satisfied only when the fixed point is bounded between the left fold point of the critical manifold and the Hopf bifurcation value, whose role will be explained in a moment. That is, when the timescale parameter, taken as bifurcation parameter, is not too small and the fixed point sits not too far from the fold point. The fixed point then loses its stability through a subcritical Hopf bifurcation for $\varepsilon \rightarrow 0$.

When the fixed point is located on the unstable part of the critical manifold (i.e., to the right of the fold point of this manifold), this allows trajectories moving on the left stable part of the manifold to freely attain and then jump at the fold point leading to a limit cycle solution. On the other hand, since the fixed point is also stable for a suitable range of $\varepsilon$, choosing an initial condition in the region of the unstable manifold near the fixed point will lead to a non-oscillatory solution. Therefore, due to the slow-fast structure of the FHN model and the very specific parameter setting, a bistable regime (necessary for ISR) consisting of a fixed point and limit cycle is established. See Fig. 3b.

Adding noise to the system in this bistable state can cause ISR. That is a switch from one basin of attraction to the other leading to a non-monotonic behavior of the number of oscillations as a function of noise strength, with a minimum at some specific noise amplitude. More specifically, when the slow dynamics moves along the stable part of the critical manifold, noise may move the trajectory into the basin of attraction of the stable fixed point, before the fold point is reached from where the solution would jump, that is, the neuron would spike. When the noise is too small, this detachment comes too late and occurs below that basin, and when the noise is too large, it comes too early and happens above that basin. But for the right noise strength, the dynamics lands in the basin of attraction of the fixed point and stays there, terminating the rhythmic spiking of the neuron.

## Model equation and phenomenon

We now consider a stochastic perturbation of the FHN neuron model Eq. (1) (FitzHugh 1961). We consider the resulting stochastic differential equation both in the slow timescale $\tau$ (Eq. (2)) and in the fast timescale *t* (Eq. (3))

2 $$\begin{aligned}&{\begin{matrix} \left\{ \begin{array}{lcl} \hbox {d}v_{\tau }&{}=&{}\frac{1}{\varepsilon }f(v_{\tau },w_{\tau })\hbox {d}\tau +\frac{\sigma }{\sqrt{\varepsilon }}\hbox {d}W_{\tau },\\ \hbox {d}w_{\tau }&{}=&{}g(v_{\tau },w_{\tau })\hbox {d}\tau , \end{array}\right. \end{matrix}} \end{aligned}$$

3 $$\begin{aligned}&{\begin{matrix} \left\{ \begin{array}{lcl} \hbox {d}v_{t}&{}=&{}f(v_{t},w_{t})\hbox {d}t+\sigma \hbox {d}W_{t},\\ \hbox {d}w_{t}&{}=&{}\varepsilon g(v_{t},w_{t})\hbox {d}t, \end{array}\right. \end{matrix}} \end{aligned}$$

with the deterministic velocity vector fields given by

4 $$\begin{aligned} {\begin{matrix} \left\{ \begin{array}{lcl} f(v,w)&{}=&{}v(a-v)(v-1)-w, \\ g(v,w)&{}=&{}bv-cw , \end{array}\right. \end{matrix}} \end{aligned}$$

where $(v,w)\in {\mathbb {R}}^2$ represent the activity of the action potential *v* and the recovery current *w* that restores the resting state of the model. We have as constant parameters $b>0$, $c>0$, and *a* is often confined to the range $0<a<1$, but the case $a < 0$ will be examined in this work.

We have a singular parameter, $0<\varepsilon :=\tau /t\ll 1$, i.e., the timescale separation ratio between the slow timescale $\tau$ and the fast timescale *t*. We note that Eq. (2) preserves the sense of the dynamics on the trajectories of Eq. (3). In other words, the phase trajectories of both systems of dynamical equations have exactly the same dynamical behavior. The only difference is the speed of these trajectories in the phase space. Because the speeds of the trajectories do not affect in any way our analysis, we will work on both timescales. The slow timescale equation at some points allows for quicker conclusions in bifurcation analysis while the fast timescale equation has an advantage in numerical simulations as it avoids the division by the very small parameter $\varepsilon$.

$$dW_t$$ is standard white noise, the formal derivative of Brownian motion with mean zero and unit variance, and $$\sigma $$ is the amplitude of this noise. The random term in Eq. (2) is rescaled in Eq. (3) according to the scaling law of Brownian motion. That is, if $$ W_t$$ is a standard Brownian motion, then for every $$\lambda >0$$, $$\lambda ^{-1/2} W_{\lambda t}$$ is also a standard Brownian motion, i.e., the two processes have the same distribution (Durrett 1996).

**Fig. 2:**
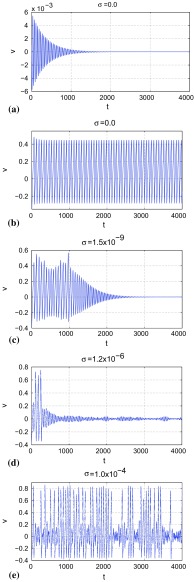
Time series of the action potential variable *v* in Eq. (3). **(a)** and **(b)** The zero-noise dynamics with initial condition in **(a)** at $\big (v(0),w(0)\big )=(0.001,0.001)$ and in **(b)** at $\big (v(0),w(0)\big )=(-\,0.4,0.2)$. **(c)**, **(d)**, and **(e)** The effects of noise of various intensities, $\sigma$, on the dynamics of *v* with $\big (v(0),w(0)\big )=(-\,0.4,0.2)$ in each case. $a=-\,0.05, b=1.0, c=2.0, \varepsilon =0.02785$

Figure 2 shows the time series produced by the dynamics of the action potential variable *v*. In the deterministic case ($\sigma =0$), and for $a=-\,0.05$, $b=1.0$, $c=2.0$, and $\varepsilon =0.02785$, Eq. (3) can result in two different dynamics. In Fig. 2a with initial conditions at $\big (v(0),w(0)\big )=(0.001,0.001)$, the neuron exhibits only subthreshold oscillations with *v* converging to zero and remaining at this value as the time *t* increases. In Fig. 2b with initial conditions now at $\big (v(0),w(0)\big )=(-\,0.4,0.2)$, the neuron now exhibits self-sustained supra-threshold oscillations. Thus, the system is bistable.

Figure2c–e shows a stochastic behavior ($\sigma >0$), with again $a=-\,0.05$, $b=1.0$, $c=2.0$, $\varepsilon =0.02785$, and the initial conditions all at $\big (v(0),w(0)\big )=(-\,0.4,0.2)$. We count a spike when *v* is greater than or equal to the threshold value $v_{th}=0.25$. In Fig. 2c with a weak-noise amplitude, i.e., $\sigma =1.5\times 10^{-9}$, we have supra-threshold oscillations for a certain time length with 21 spikes after which *v* starts to converge to zero and the spiking eventually stops. In Fig. 2d, when the noise amplitude is increased (but still relatively weak) to $\sigma =1.2\times 10^{-6}$, we have an even faster inhibition of the spiking with a smaller number of spikes. In this realization, we have only 3 spikes. In Fig. 2e, with a stronger noise amplitude, $\sigma =1.0\times 10^{-4}$, the number of spikes increases again up to 38. Intuitively, it is surprising that weak-noise amplitudes inhibit the spiking activity of the neuron with the occurrence of a minimum in the number of spikes as the noise amplitude increases even though the initial conditions are exactly the same as in Fig. 2b. We shall investigate in detail the mechanisms behind this phenomenon.

## Deterministic bifurcation analysis

We now consider the deterministic dynamics corresponding to Eq. (2) when $\sigma = 0$ and perform a bifurcation analysis through which we find the parametric conditions necessary for a subcritical Hopf bifurcation and a bistable regime consisting of a unique stable fixed point and a stable limit cycle. In this section, for the sake of briefness and clarity, we will restrict our analysis to just the relevant fixed point and regime of parameters leading to bi-stability. For a complete and explicit bifurcation and slow-fast analysis of the model in Eq. (2), we refer the reader to Appendices.

The *v*-nullcline (denoted by ${\mathcal {M}}_0$) of Eq. (2) is defined as ${\mathcal {M}}_0:w=-\,v^3+(a+1)v^2-av$. ${\mathcal {M}}_0$ changes its stability at its extrema, the so-called singular points (or fold points), both located at $v_\pm = \frac{a+1}{3} \pm \frac{1}{3}\sqrt{a^2-a+1}$. This stability property follows from the fact that $\frac{\hbox {d}w}{\hbox {d}\tau }$ is positive on the ascending branch of ${\mathcal {M}}_0$: $v_{0}^*(w)={\mathcal {M}}_0\cap \{v_{-}\le v \le v_+\}$, which hence is unstable, and negative on the two descending branches of ${\mathcal {M}}_0$: $v_\pm ^*(w)={\mathcal {M}}_0\cap \{\pm v> \pm v_\pm \}$, which are stable. We also note here that $v_-<0$ if and only if $a<0$.

${\mathcal {M}}_0$ intersects the *w*-nullcline ($w=\frac{b}{c}v$) of Eq. (2) at one, two or three different fixed points (the rest states of the neuron). One of them is $(v_0,w_0)=(0,0)$, and we assume for the purpose of this paper that this is the only fixed point (see “Appendices”). Standard analysis shows that $v_0$ is stable when $-\frac{a}{\varepsilon }<c$ (and $a>-\frac{b}{c})$, that is, when *a* is not too negative, and in the limit $\varepsilon \rightarrow 0$ only for $a\ge 0$. Moreover, when $c^2 <\frac{b}{\varepsilon }$ and $3\varepsilon c \le a^2-a +1$, we get a Hopf bifurcation at

5 $$\begin{aligned} v_{\star }=\frac{a+1}{3}-\sqrt{\frac{(a+1)^2}{9}-\frac{a+\varepsilon c}{3}}. \end{aligned}$$

For $a<0$, $v_-<v_0=0$, and this Hopf bifurcation occurs at $v_0=0$ for the value

6 $$\begin{aligned} \varepsilon _{hp}=-\,\frac{a}{c}. \end{aligned}$$

Thus, $v_0$ loses its stability through a subcritical Hopf bifurcation when $\varepsilon$ decreases. As long as $-\frac{a}{c}<\varepsilon$, we have a *stable* fixed point $(v_0,w_0)=(0,0)$ to the right of the fold point at $v_-=\frac{a+1}{3} - \sqrt{\frac{(a+1)^2}{9}-\frac{a}{3}}$. We choose and maintain throughout this work the values of the parameters as: $a=-\,0.05$, $b=1.0$, and $c=2.0$. For these values, we have $v_-=-\,0.25305<v_0=0<-\frac{a}{c}=0.025$ and therefore $v_0$ is located on the middle part of ${\mathcal {M}}_0$ and it is stable. The Hopf bifurcation value of $\varepsilon$ is computed from Eq. (6) as $\varepsilon _{hp}=0.025$.

**Fig. 3:**
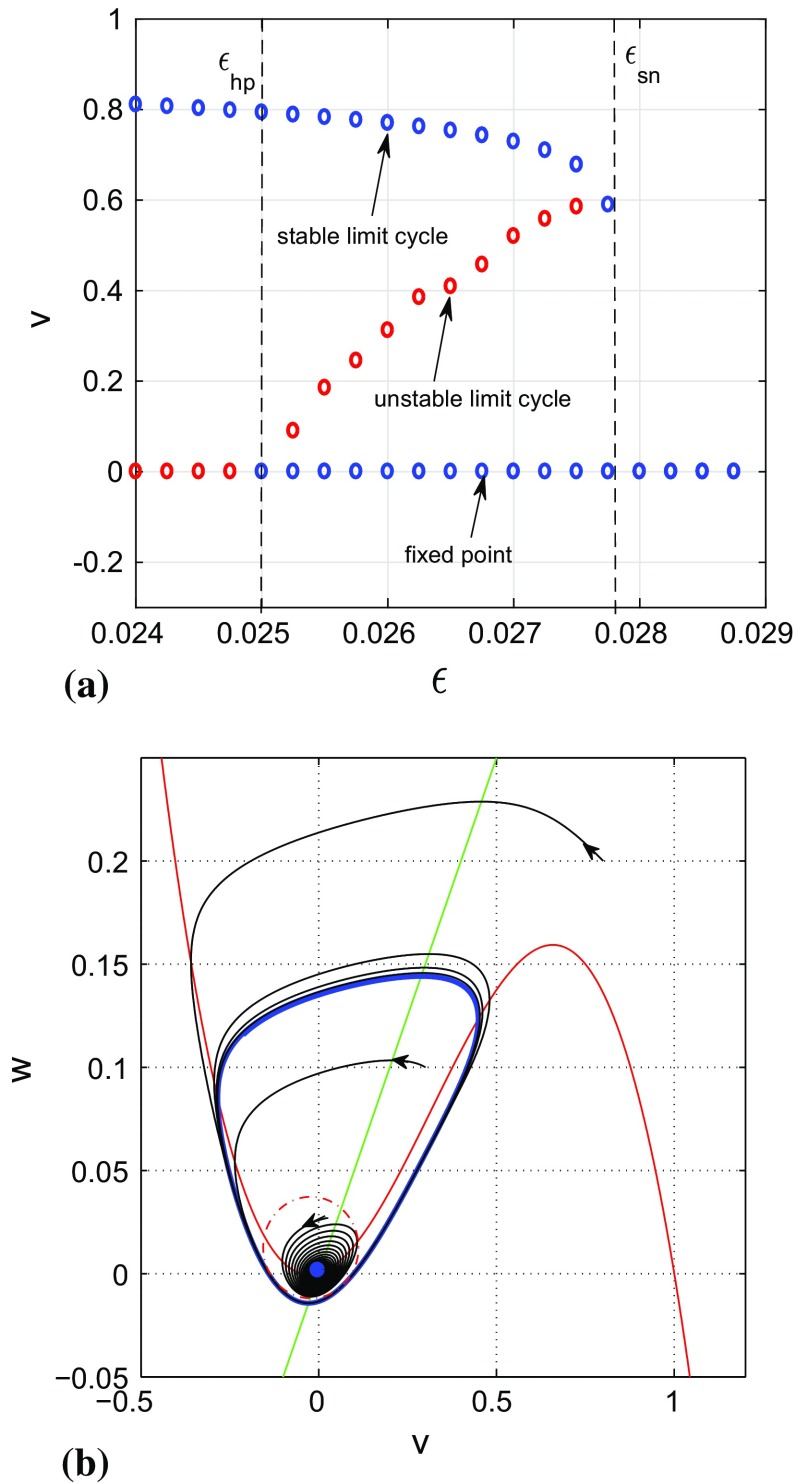
**(a)** Bifurcation diagram for Eq. (3) with a fixed point at $(v_0,w_0)=(0,0)$ unstable in the singular parameter range $0.024<\varepsilon <0.025$ shown by the red points and a stable limit cycle in this same interval. At $\varepsilon =\varepsilon _{hp}=0.025$, $(v_0,w_0)$ gain stability through a subcritical Hopf bifurcation and therefore coexist with the stable limit in the interval $0.025<\varepsilon <0.027865$. The stable limit cycle undergoes a saddle-node bifurcation and disappears by shrinking and eventually colliding with the boundary of the basin of attraction (the unstable limit cycle represented by the red dots between the fixed point and stable limit cycle) of the stable fixed point at $\varepsilon =\varepsilon _{sn}=0.027865$. In $0.027865<\varepsilon \le 0.029$, there is only the stable fixed point $(v_0,w_0)$ in the entire phase space. **(b)** Geometry of attractors of Eq. (3) with $\varepsilon _{hp}<\varepsilon <\varepsilon _{sn}$. The red curve represents the cubic critical manifold ${\mathcal {M}}_0$ intersecting the *w*-nullcline (the green line) at the blue dot corresponding to the fixed point $(v_0,w_0)=(0,0)$, located to the right of the minimum of ${\mathcal {M}}_0$ at $v_-=-\,0.25305$. The blue closed curve represents the stable limit cycle, the red dotted closed curve the pseudo-separatrix (the unstable limit cycle), and 3 different trajectories (in black) with arrows at the initial conditions. Depending on which side of the separatrix the initial conditions are chosen, solutions converge to either the stable limit cycle or to the stable fixed point. $a=-\,0.05, b=1.0, c=2.0, \varepsilon =0.02785, \sigma =0.0$

For these values of the system parameters, see Fig. 3a, we computed the bifurcation diagram by selecting the maximum values of the action potentials *v* as a function of the bifurcation parameter $\varepsilon$, for both the stable and unstable limit cycles. For $0.024\le \varepsilon <0.025$, the fixed point $v_0=0$ is unstable as $\mathrm {det}J_{ij}=\frac{0.9}{\varepsilon }>0$ and $\mathrm {tr}J_{ij}=\frac{0.05}{\varepsilon }-2>0$ and surrounded by a stable limit cycle, and then no bi-stability occurs. At $\varepsilon =\varepsilon _{hp}=0.025$, a subcritical Hopf bifurcation occurs and the unstable fixed point $v_0=0$ changes its stability. For $0.025<\varepsilon <0.027865$, the fixed point $v_0=0$ is stable ($\mathrm {det}J_{ij}>0$ and $\mathrm {tr}J_{ij}<0$ for those values of $\varepsilon$) and coexists with the stable limit cycle. Thus, for $0.025<\varepsilon <0.027865$ we have a bi-stability regime. At $\varepsilon =\varepsilon _{sn}=0.027865$, we have a saddle-node bifurcation of limit cycles, in which case the stable limit cycle surrounding the stable fixed point $v_0=0$ shrinks and eventually collides with the boundary of the basin of attraction of $v_0$ (i.e., the unstable limit cycle). In this saddle-node bifurcation, the stable and the unstable limit cycle annihilate each other leaving behind the stable fixed point $v_0$. The fixed point $v_0$ maintains its stability in the interval $0.027865<\varepsilon \le 0.029$, within which we have no bi-stability as there is only one attractor in the entire phase space.

In the bi-stability regime $\varepsilon _{hp}<\varepsilon <\varepsilon _{sn}$, depending on whether the initial conditions are chosen in the basin of attraction of the fixed point or in that of the limit cycle, the dynamics will converge to either the fixed point or to the limit cycle. This behavior is seen in Fig. 3b (also already seen in the time series in Fig. 2a,b) which shows a phase portrait of one trajectory with initial conditions in the basin of attraction of the stable fixed point at $(v_0,w_0)=(0,0)$ (the blue dot at the origin) and two other trajectories with initial conditions in the basin of attraction of the stable limit cycle (the blue closed curve). In this work, we will focus on weak-noise effects on the spiking dynamics of Eq. (2) with $\varepsilon _{hp}<\varepsilon <\varepsilon _{sn}$.

## Stochastic sensitivity analysis and the Mahalanobis metric

We now introduce noise to the neuron model (i.e., $\sigma >0$) and perform a stochastic sensitivity analysis of the stable attractors. In this section, we analyze the neuron model on the fast timescale *t*. The stochastic sensitivity matrix associated to a stochastic dynamical system is an asymptotic characteristic of the random attractors of the system (Bashkirtseva and Ryashko 2004). For our model equation Eq. (3) with $0<\sigma \ll 1$, it allows us to approximate a spread of random trajectories around the stable fixed point $(v_0,w_0)=(0,0)$ and stable limit cycle which we now denote by $\big [\bar{v}(t),\bar{w}(t)\big ]$. The random trajectories in the basin of attraction of the stable fixed point $(v_0,w_0)$ evolve according to the evolution of the probability density of the FPE corresponding to Eq. (3) (Risken 1989).

Suppose that a stationary solution, $P\big [v(t),w(t)\big ]$, of this FPE exists. Generally, for *n*-dimensional systems with $n\ge 2$, one usually cannot find such a stationary probability density analytically (Risken 1989). This is the situation with Eq. (3). When $0<\sigma \ll 1$, the constructive asymptotics and approximations based on a quasi-potential function, $\varphi$, given in Eq. (7) are frequently used (Freidlin and Wentzell 1998).

7 $$\begin{aligned} \varphi =-\,\lim _{\sigma \rightarrow 0}\sigma ^2\log P\Big \{\big [v(t),w(t)\big ],\sigma \Big \}. \end{aligned}$$

A quadratic form of the quasi-potential gives a Gaussian approximation of $P_g\big [v(t),w(t)\big ]$ in the vicinity of the fixed point $(v_0,w_0)$,

8 $$\begin{aligned}&P_g\Big \{\big [v(t),w(t)\big ];(v_0,w_0)\Big \}\nonumber \\&\quad =\frac{1}{Z} \exp \Bigg [-\frac{1}{2\sigma ^2} \left( \begin{array}{c} v(t)-v_0\\ w(t)-w_0 \end{array}\right) ^{\top }\varOmega _{ij}^{-1} \left( \begin{array}{c} v(t)-v_0\\ w(t)-w_0 \end{array}\right) \Bigg ], \end{aligned}$$

where *Z* is the normalization constant and $\varOmega _{ij}$ is the covariance matrix of random trajectories around the stable fixed point $(v_0,w_0)$, i.e., $\varOmega _{ij}$ plays the role of the stochastic sensitivity matrix of this stable fixed point and it is determined by the algebraic equation

9 $$\begin{aligned} J_{ij}\varOmega _{ij}+\varOmega _{ij}J_{ij}^{\top }+G_{ij}=\mathbf 0 , \end{aligned}$$

$$J_{ij}=\left( \begin{array}{cc} -\,a \,\,\,&{} -\,1\\ \varepsilon b \,\,\,&{} -\,\varepsilon c \end{array} \right) $$ is the Jacobian matrix at $(v_0,w_0)=(0,0)$ of the deterministic neuron equation Eq. (3). $$G_{ij}=\left( \begin{array}{cc} 1 &{} 0\\ 0 &{} 0 \end{array} \right) $$ is the diffusion matrix of system Eq. (3) and $\top$ denotes the transpose.

As the fixed point $(v_0,w_0)$ is exponentially stable (that is, all the eigenvalues of $J_{ij}$ at $(v_0,w_0)=(0,0)$ have strictly negative real parts), the matrix equation in Eq. (9) has as its unique solution the stochastic sensitivity matrix $\varOmega _{ij}\overline{}$ of the fixed point (Bashkirtseva and Ryashko 2004). The eigenvalues $\lambda _k(\varepsilon )$, $k=\{1,2\}$ of the stochastic sensitivity matrix $\varOmega _{ij}$ define the variance of the random trajectories around the fixed point $(v_0,w_0)$. The largest eigenvalue (largest SSF) $\lambda _{max}=\text {max}\{\lambda _k(\varepsilon )\}$ for each value of the singular parameter $\varepsilon$ indicates the sensitivity of the stable fixed point $(v_0,w_0)$ to the random perturbation. As $\lambda _{max}$ increases, the sensitivity of the $(v_0,w_0)$ to noise also increases. This means we have a higher probability of escape (i.e., shorter residence time) from the basin of attraction of the stable fixed point $(v_0,w_0)$ which we now denote for short as $\mathcal {B}(v_0,w_0)$.

The matrix equation Eq. (9) for Eq. (3) reduces to the system of algebraic equations

10 $$\begin{aligned} {\begin{matrix} \left\{ \begin{array}{lcl} -2a\varOmega _{11}-\varOmega _{12}-\varOmega _{21}+1=0,\\ b\varepsilon \varOmega _{11}+(-\,a-c\varepsilon )\varOmega _{12}-\varOmega _{22}=0,\\ b\varepsilon \varOmega _{11}+(-\,c\varepsilon -a)\varOmega _{21}-\varOmega _{22}=0,\\ b\varepsilon \varOmega _{12}+b\varepsilon \varOmega _{21}-2c\varepsilon \varOmega _{22}=0. \end{array}\right. \end{matrix}} \end{aligned}$$

In the parametric zone of bi-stability: $a=-\,0.05$, $b=1.0$, $c=2.0$, $\varepsilon _{hp}<\varepsilon <\varepsilon _{sn}$, the stochastic sensitivity matrix $\varOmega _{ij}$ of the stable fixed point at $(v_0,w_0)=(0,0)$ of Eq. (3) is given by

11 $$\begin{aligned} \varOmega _{ij}=\varOmega _{ji}=\left( \begin{array}{cc} \frac{4\varepsilon +0.9}{3.6\varepsilon -0.09}\,\,\,\,\,&{} \frac{\varepsilon }{1.8\varepsilon -0.045}\\ \\ \frac{\varepsilon }{1.8\varepsilon -0.045}\,\,\,\,\,&{} \frac{\varepsilon }{3.6\varepsilon -0.09} \end{array} \right) , \end{aligned}$$

with the eigenvalues (the SSFs) given by

12 $$\begin{aligned} \lambda _{1,2}(\varepsilon )=\frac{5\varepsilon + 0.9\mp \sqrt{25\varepsilon ^2+5.4\varepsilon +0.81}}{7.2\varepsilon -0.18}, \end{aligned}$$

where $\lambda _{max}=\lambda _2(\varepsilon )$ . The corresponding generalized eigenvectors are

13 $$\begin{aligned} U_{1,2}(\varepsilon )=\left( \begin{array}{c} \frac{0.04\varepsilon }{-0.3\varepsilon -0.9\mp \sqrt{25\varepsilon ^2+5.4\varepsilon +0.81}} \\ \\ 0.05 \end{array} \right) . \end{aligned}$$

**Fig. 4:**
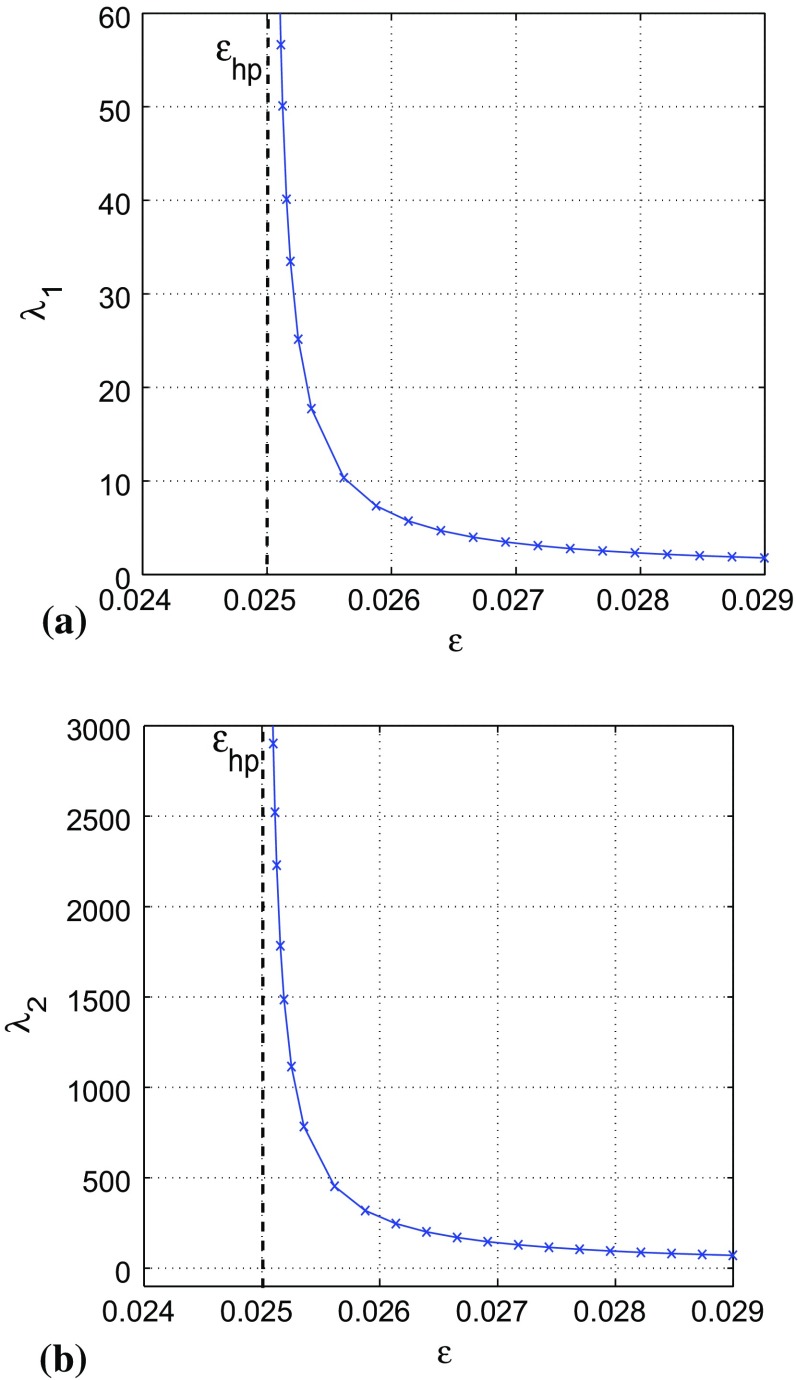
Variation of the SSFs $\lambda _1$ in **(a)** and $\lambda _2$ in **(b)** of the stable fixed point at $(v_0,w_0)=(0,0)$ with the singular parameter $\varepsilon$. The SSFs diverge at the Hopf bifurcation value at $\varepsilon =\varepsilon _{hp}=0.025$, with $\lambda _2$ dominating $\lambda _1$, indicating a higher stochastic sensitivity of the fixed point in the direction of the corresponding eigenvector $U_2$

For a fixed noise strength $\sigma$, the difference between $\lambda _1$ and $\lambda _2$ reflects a spatial non-uniformity of the dispersion of the random trajectories around the fixed point $(v_0,w_0)$ in the direction of the eigenvectors $U_1$ and $U_2$, respectively. The dependence of $\lambda _1$ and $\lambda _2$ on the singular parameter $\varepsilon$ is shown in Fig. 4.

Firstly, we observe that the SSFs diverge as we approach the Hopf bifurcation value at $\varepsilon =\varepsilon _{hp}=0.025$. This means that the fixed point $(v_0,w_0)$ becomes more and more sensitive to noise as we approach the Hopf bifurcation value and therefore the highest probability of escape (i.e., shortest residence time) from $\mathcal {B}(v_0,w_0)$ when $\varepsilon \approx \varepsilon _{hp}$.

Secondly, we observe that $\lambda _2$ diverges faster than $\lambda _1$ as $\varepsilon \rightarrow \varepsilon _{hp}=0.025$. This shows that the eigenvector $U_2$ localizes the main direction for deviations of random trajectories from the fixed point $(v_0,w_0)$, providing the direction in which the intersection with the unstable limit cycle at [*v*(*t*), *w*(*t*)] is most probable.

The Mahalanobis metric is a widely used metric in cluster and discriminant analyses (McLachlan 2004). Basically, it measures the distance between a point *x* and a distribution. This metric is a natural tool for the quantitative analysis of noise-induced transitions as it combines both the geometric distance from a random attractor to a point and the stochastic sensitivity of this attractor. The metric allows us to estimate a preference of the stable fixed point $(v_0,w_0)$ or the stable limit cycle $\big [\bar{v}(t),\bar{w}(t)\big ]$ in the stochastic dynamics of Eq. (3), when the random trajectory passes from one attractor to another. For $0<\sigma \ll 1$, the Mahalanobis distance from the unstable limit cycle $\big [v(t),w(t)\big ]$ (separatrix) to the stable fixed point $(v_0,w_0)$ or to the stable limit cycle $\big [\bar{v}(t),\bar{w}(t)\big ]$ is related to the residence time of trajectories in the corresponding basin of attraction: the larger the Mahalanobis distance, the longer is the residence time (i.e., lower probability of escape) in the corresponding basin.

In the stochastic sensitivity analysis of our fixed point $(v_0,w_0)$, we approximate the probability density by a Gaussian distribution in Eq. (8) centered at the stable fixed point at $(v_0,w_0)=(0,0)$. The Mahalanobis distance $D_m\Big \{\big [v(t),w(t)\big ];(v_0,w_0)\Big \}$ from a point $\big [v(t),w(t)\big ]$ (i.e., a point on the unstable limit cycle) to the distribution of random trajectories around the stable attractor at $(v_0,w_0)$ is given by

14 $$\begin{aligned}&D_m\Big \{\big [v(t),w(t)\big ];(v_0,w_0)\Big \}\nonumber \\&\quad =\sqrt{\left( \begin{array}{c} v(t)-v_0\\ w(t)-w_0 \end{array}\right) ^{\top }\varOmega _{ij}^{-1}\left( \begin{array}{c} v(t)-v_0\\ w(t)-w_0 \end{array}\right) }, \end{aligned}$$

where $\varOmega _{ij}$ is the stochastic sensitivity matrix of the fixed point $(v_0,w_0)$, and so the Gaussian approximation in Eq. (8) can be written in terms of the Mahalanobis distance as

15 $$\begin{aligned}&P_g\Big \{\big [v(t),w(t)\big ];(v_0,w_0)\Big \}\nonumber \\&\quad =\frac{1}{Z}\exp \left[ -\frac{\bigg (D_m\Big \{\big [v(t), w(t)\big ];(v_0,w_0)\Big \}\bigg )^2}{2\sigma ^2}\right] . \end{aligned}$$

For Eq. (3), we calculate the Mahalanobis distances from the stable fixed point at $(v_0,w_0)=(0,0)$ to points on the unstable limit cycle at $\big [v(t),w(t)\big ]$, and then we choose the minimal distance. We calculate coordinates of the unstable limit cycle numerically. This is done by assigning $\big [v(t),w(t)\big ]$ to the *limiting values* of the initial conditions $\big (v(0),w(0)\big )$ such that infinitesimal perturbations (to the right and to the left) of these initial conditions will lead to the convergence of the trajectories either to the stable fixed point at $(v_0,w_0)$ or to the stable limit cycle at $\big [\bar{v}(t),\bar{w}(t)\big ]$ depending on which side the infinitesimal perturbation is made.

We have

16 $$\begin{aligned} \varOmega _{ij}^{-1}=\left( \begin{array}{cc} 4\varepsilon -0.1 \,\,\,\,\,&{} -8\varepsilon +0.2\\ \\ -8\varepsilon +0.2 \,\,\,\,\,&{} \frac{16\varepsilon ^2+3.2\varepsilon -0.09}{\varepsilon } \end{array} \right) , \end{aligned}$$

and using Eq. (14), we calculate the Mahalanobis distance from the stable fixed $(v_0,w_0)=(0,0)$ to the unstable limit cycle $\big [v(t),w(t)\big ]$ as

17 $$\begin{aligned} D_m\Big \{\big [v(t),w(t)\big ];(0,0)\Big \}&=\Bigg [ \frac{16\varepsilon ^2+3.2\varepsilon -0.09}{\varepsilon }w(t)^2\nonumber \\&\quad +(0.4-16\varepsilon )v(t)w(t)\nonumber \\&\quad +(4\varepsilon -0.1)v(t)^2\Bigg ]^{1/2}. \end{aligned}$$

Because of the dominance of $\lambda _2$ over $\lambda _1$, we numerically calculate the minimum Mahalanobis distance $D_m$ from the fixed point at $(v_0,w_0)=(0,0)$ to all points on the unstable limit cycle at $\big [v(t),w(t)\big ]$ by approximating the Mahalanobis distance in Eq. (17) along the eigenvector $U_2$, i.e.,

18 $$\begin{aligned} D_m=\min _{(v,w)\in \big [v(t),w(t)\big ]}D_m\Big \{\big [v(t),w(t)\big ] ;(0,0)\Big \}. \end{aligned}$$

**Fig. 5:**
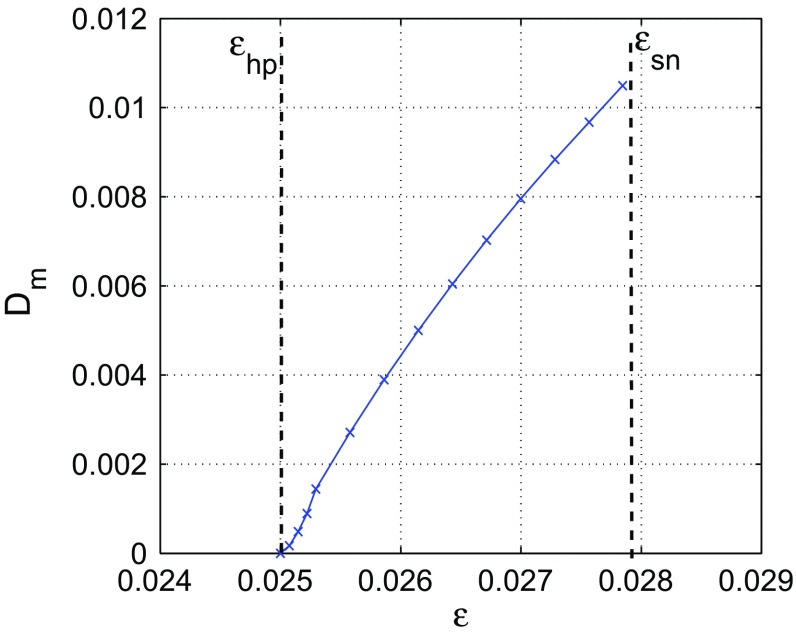
Minimal Mahalanobis distance $D_m$ from the stable fixed point at $(v_0,w_0)=(0,0)$ to the unstable limit cycle at $\big [v(t),w(t)\big ]$. Vertical dashed lines show the location of the subcritical Hopf bifurcation of stable fixed point at $\varepsilon _{hp}=0.025$ and of the saddle-node bifurcation of the stable limit cycle at $\varepsilon _{sn}=0.027865$. $D_m$ vanishes at $\varepsilon =\varepsilon _{hp}$ and has maximum value just before $\varepsilon =\varepsilon _{sn}$. $a=-\,0.05, b=1.0, c=2.0$

Figure 5 shows the variation of the minimal Mahalanobis distance $D_m$ from the fixed point $(v_0,w_0)$ to the unstable limit cycle with the singular parameter $\varepsilon$. The Mahalanobis distance vanishes at the subcritical Hopf bifurcation value $\varepsilon _{hp}=0.025$ and increases with increasing $\varepsilon$. This means as $\varepsilon$ increases from $\varepsilon _{hp}$, the basin of attraction of the fixed point increases in the direction of the eigenvector $U_2$, and therefore a lower and lower probability of escape (i.e., longer residence time) from $\mathcal {B}(v_0,w_0)$ results.

From Figs. 4b and 5, we see that the two factors (stochastic sensitivity of an attractor and distance of the attractor to the separatrix) determining the length of the residence time of random trajectories in $\mathcal {B}(v_0,w_0)$ are not competing. Approaching $\varepsilon _{hp}$ from above increases the SSF of the fixed point and at the same time decreases its Mahalanobis distance to the unstable limit cycle. This has the combined effect of considerably reducing the residence time of trajectories in $\mathcal {B}(v_0,w_0)$. In other words, there is a higher probability (lower probability) that the random trajectories escape from $\mathcal {B}(v_0,w_0)$ when $\varepsilon \rightarrow \varepsilon _{hp}$ ($\varepsilon \rightarrow \varepsilon _{sn}$).

We now apply a similar analysis to our randomly perturbed stable limit cycle. In the deterministic system ($\sigma =0$) of Eq. (3), with $\varepsilon _{hp}<\varepsilon <\varepsilon _{sn}$, we have an exponentially stable limit cycle defined by a *T*-periodic solution, $\big [\bar{v}(t),\bar{w}(t)\big ]=\big [\bar{v}(t+T),\bar{w}(t+T)\big ]$. For the transversal hyperplane $\varSigma _{t}$ in the neighborhood of any point $\big [\bar{v}(t),\bar{w}(t)\big ]$ on the stable limit cycle, the Gaussian approximation of the probability density reads

19 $$\begin{aligned}&P_g\Big \{\big [v(t),w(t)\big ];[\bar{v}(t),\bar{w}(t)\big ]\Big \}\nonumber \\&\quad =\frac{1}{Z}\exp \Bigg [-\frac{1}{2\sigma ^2} \left( \begin{array}{c} v(t)-\bar{v}(t)\\ w(t)-\bar{w}(t) \end{array}\right) ^{\top }\varTheta _{ij}^{-1}(t)\nonumber \\&\qquad \times \left( \begin{array}{c} v(t)-\bar{v}(t)\\ w(t)-\bar{w}(t) \end{array}\right) \Bigg ]. \end{aligned}$$

Here, the stochastic sensitivity matrix is periodic in time, $\varTheta _{ij}(t)=\varTheta _{ij}(t+T)$. For an exponentially stable limit cycle, the largest Lyapunov exponent is 0 and the others are negative. Consequently, the matrix $\varTheta _{ij}(t)$ is the unique solution of the Lyapunov equation (Bashkirtseva and Ryashko 2004),

$$\begin{aligned} \frac{d\varTheta _{ij}}{\hbox {d}t}&= J_{ij}(t)\varTheta _{ij}(t)+\varTheta _{ij}(t)J_{ij}(t)^{\top }+ P_{ij}(t) G_{ij} P_{ij}(t), \end{aligned}$$

with the conditions

20 $$\begin{aligned} {\begin{matrix} \left\{ \begin{array}{lcl} \varTheta _{ij}(0)=\varTheta _{ij}(T),\\ \\ \varTheta _{ij}(t)\left( \begin{array}{c} f\big [\bar{v}(t),\bar{w}(t)\big ]\\ \\ g\big [\bar{v}(t),\bar{w}(t)\big ] \end{array} \right) \equiv 0, \end{array}\right. \end{matrix}} \end{aligned}$$

where $P_{ij}(t)$ is a matrix of the orthogonal projection onto the Poincaré section $\varSigma _{t}$ at the point $[\bar{v}(t),\bar{w}(t)]$ on the stable limit cycle, which is symmetric for our model equation Eq. (3), and whose entries are given by

21 $$\begin{aligned} {\begin{matrix} \left\{ \begin{array}{lcl} P_{11}=(-\,\bar{v}^3+(a+1)\bar{v}^2-a\bar{v}-\bar{w})^2,\\ P_{12}=\varepsilon (-\,\bar{v}^3+(a+1)\bar{v}^2-a\bar{v}-\bar{w})(b\bar{v}- c\bar{w}),\\ P_{22}=\varepsilon ^2(b\bar{v}-c\bar{w})^2. \end{array}\right. \end{matrix}} \end{aligned}$$

$J_{ij}(t)$ is the Jacobian of the deterministic neuron at a point $[\bar{v}(t),\bar{w}(t)]$ on the stable limit cycle and given by

22 $$\begin{aligned} J_{ij}(t)=\left( \begin{array}{cc} -3\bar{v}^2+2(a+1)\bar{v}-a &{}\,\,\,\,\, -1\\ \\ \varepsilon b&{}\,\,\,\,\, -\varepsilon c\end{array} \right) , \end{aligned}$$

and the constant diffusion matrix $G_{ij}$ is the same as before.

The Mahalanobis distance from a point $\big [v(t),w(t)\big ]$ on the unstable limit cycle to the distribution of random trajectories around the stable limit cycle at $\big [\bar{v}(t),\bar{w}(t)\big ]$, $D_m\Big \{\big [v(t),w(t)\big ];\big [\bar{v}(t),\bar{w}(t)\big ]\Big \}$, is also a periodic function of time and given by

23 $$\begin{aligned}&D_m\Big \{\big [v(t),w(t)\big ];\big [\bar{v}(t),\bar{w}(t)\big ]\Big \}\nonumber \\&\quad =\sqrt{\left( \begin{array}{c} v(t)-\bar{v}(t)\\ w(t)-\bar{w}(t) \end{array}\right) ^{\top }\varTheta _{ij}^{+}(t)\left( \begin{array}{c} v(t)-\bar{v}(t)\\ w(t)-\bar{w}(t) \end{array}\right) }. \end{aligned}$$

Because $\varTheta _{ij}(t)$ is singular for Eq. (3), “+” means a pseudo-inverse in this case.

For 2-D systems, $\varTheta _{ij}(t)$ can also be written in the form (Bashkirtseva and Perevalova 2007)

24 $$\begin{aligned} {\begin{matrix} \left\{ \begin{array}{lcl} \varTheta _{ij}(t)=\mu (t)P_{ij}(t),\\ \varTheta _{ij}^{+}(t)=\frac{1}{\mu (t)}P_{ij}(t), \end{array}\right. \end{matrix}} \end{aligned}$$

and the Mahalanobis distance is given by

25 $$\begin{aligned} D_m\Big \{\big [v(t),w(t)\big ];\big [\bar{v}(t),\bar{w}(t)\big ]\Big \}= \frac{\left||\left( \begin{array}{c} v(t)-\bar{v}(t)\\ w(t)-\bar{w}(t) \end{array}\right) \right||}{\sqrt{\mu (t)}}. \end{aligned}$$

Here, $\mu (t)=\mu (t+T)>0$ is the unique solution of the boundary problem

26 $$\begin{aligned} {\begin{matrix} \left\{ \begin{array}{lcl} \hbox {d}\mu =\alpha (t)\mu (t)\hbox {d}t+\beta (t)\hbox {d}t,\\ \mu (0)=\mu (T), \end{array}\right. \end{matrix}} \end{aligned}$$

with *T*-periodic coefficients

27 $$\begin{aligned} {\begin{matrix} \left\{ \begin{array}{lcl} \alpha (t)=q(t)^\top \big [J(t)^\top + J(t)\big ]q(t),\\ \beta (t)=q(t)^\top G_{ij}q(t). \end{array}\right. \end{matrix}} \end{aligned}$$

*q*(*t*) is a normalized vector orthogonal to the velocity vector field $$\left( \begin{array}{c} f\big [\bar{v}(t),\bar{w}(t)\big ] \\ g\big [\bar{v}(t),\bar{w}(t)\big ] \end{array} \right) $$ and for Eq. (3) is given by

28 $$\begin{aligned}&q(t)=\left( \begin{array}{c}-\varepsilon (b\bar{v}-c\bar{w})\\ -\bar{v}^3+(a+1)\bar{v}^2-a\bar{v}-\bar{w} \end{array} \right) \nonumber \\&\quad \times \frac{1}{\sqrt{(-\,\bar{v}^3+(a+1)\bar{v}^2-a\bar{v}-\bar{w})^2 +\varepsilon ^2(b\bar{v}-c\bar{w})^2}}. \end{aligned}$$

We note that because our model is 2-dimensional, the hyperplane given by $\varSigma _{t}$ is a tangent line to the stable limit cycle solution which is normal to *q*(*t*) at $[\bar{v}(t),\bar{w}(t)]$. The functions $\alpha (t)$ and $\beta (t)$ for Eq. (3) are given by

29 $$\begin{aligned} \alpha (t)&=\frac{1}{(-\,\bar{v}^3+(a+1)\bar{v}^2-a\bar{v}-\bar{w})^2 +\varepsilon ^2(b\bar{v}-c\bar{w})^2}\nonumber \\&\quad \times \Bigg [2\varepsilon ^2(-\,3\bar{v}^2+2(a+1)\bar{v}-a)(b\bar{v}- c\bar{w})^2\nonumber \\&\quad -2\varepsilon (\varepsilon b-1)(b\bar{v}-c\bar{w})(-\,\bar{v}^3+(a+1) \bar{v}^2-a\bar{v}-\bar{w})\nonumber \\&\quad -2\varepsilon c(-\,\bar{v}^3+(a+1)\bar{v}^2-a\bar{v}-\bar{w})^2\Bigg ]. \end{aligned}$$

30 $$\begin{aligned} \beta (t)&=\frac{\varepsilon ^2(b\bar{v}-c\bar{w})^2}{(-\,\bar{v}^3+(a+1) \bar{v}^2-a\bar{v}-\bar{w})^2+\varepsilon ^2(b\bar{v}-c\bar{w})^2}. \end{aligned}$$

The explicit solution of Eq. (26) is given by

31 $$\begin{aligned} \mu (t)=e^{\int _0^t\alpha (s)ds}\Bigg [\int _0^t \beta (s)e^{\int _0^s-\alpha (r)dr}ds+C\Bigg ]. \end{aligned}$$

Because $\mu (t)$ is *T*-periodic, we write

32 $$\begin{aligned}&e^{\int _0^t\alpha (s)ds}\Bigg [\int _0^t \beta (s)e^{\int _0^s-\alpha (r)dr}ds+C\Bigg ]\nonumber \\&\quad =e^{\int _0^{t+T}\alpha (s)ds}\Bigg [\int _0^{t+T} \beta (s)e^{\int _0^s-\alpha (r)dr}ds+C\Bigg ]\nonumber \\&\quad =e^{\int _0^{t}\alpha (s)ds}e^{\int _t^{t+T}\alpha (s)ds} \Bigg [\int _0^{t}\beta (s)e^{\int _0^s-\alpha (r)dr}ds\nonumber \\&\qquad +\int _t^{t+T}\beta (s)e^{\int _0^s-\alpha (r)dr}ds+C \Bigg ], \end{aligned}$$

and use the periodic property: $\alpha (t)=\alpha (t+T)$ with $\int _t^{t+T}\alpha (s)ds=\int _0^T\alpha (t+s)ds$, for a fixed *t*, to get the constant *C* as

33 $$\begin{aligned} C=\frac{e^{\int _{0}^{T}\alpha (s)ds}\cdot \int _{0}^{T}\beta (s)e^{\int _{0}^{s}-\alpha (r)dr}ds}{1-e^{\int _{0}^{T}\alpha (s)ds}}. \end{aligned}$$

**Fig. 6:**
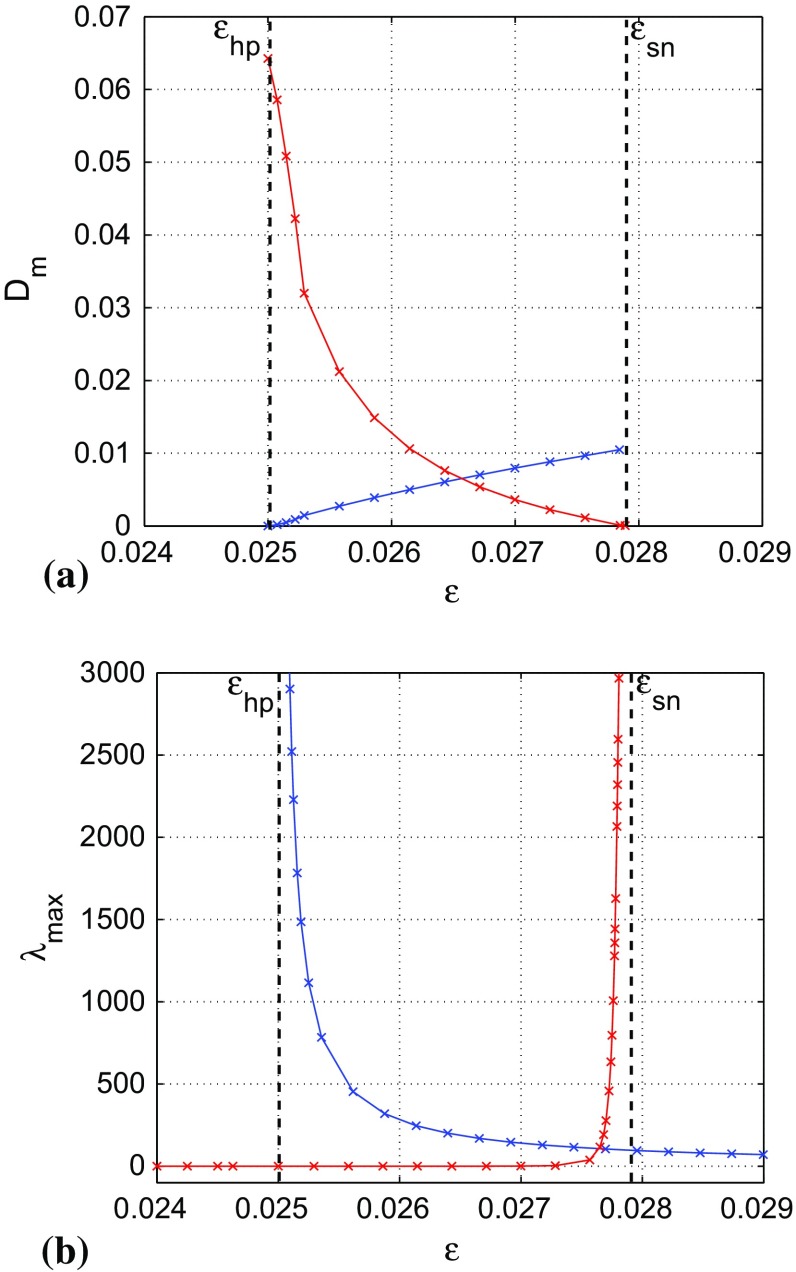
Variations of minimal Mahalanobis distances and the SSFs of the attractors for Eq. (3) with the singular parameter $\varepsilon$. In **(a)**, the blue curve shows the minimal Mahalanobis distance, $D_m$, between stable fixed point at $(v_0,w_0)=(0,0)$ and the unstable limit cycle at [*v*(*t*), *w*(*t*)] and the red curve corresponds to the minimal Mahalanobis distance between stable limit cycle at $\big [\bar{v}(t),\bar{w}(t)\big ]$ and the unstable limit cycle, with $D_m$ vanishing at $\varepsilon _{sn}$. **(b)** The SSFs of the stable fixed point (blue curve) and that of the stable limit cycle (red curve). $a=-\,0.05, b=1.0, c=2.0$

With Eq. (25) and the numerical value of $\mu (t)$ in Eq. (31), the Mahalanobis distance is computed as in Eq. (34) and the minimal Mahalanobis distance is calculated by taking the minimum value of Eq. (34) over $t\in [0,T)$ and $(v,w)\in [v(t),w(t)]$. See Fig. 6a.

34 $$\begin{aligned}&D_m\Big \{\big [v(t),w(t)\big ];\big [\bar{v}(t),\bar{w}(t)\big ]\Big \}\nonumber \\&\quad =\sqrt{\frac{(v(t)-\bar{v}(t))^2+(w(t)-\bar{w}(t))^2}{\mu (t)}}. \end{aligned}$$

This set, we obtain the entries of $\varTheta _{ij}(t)$ in Eq. (24) using the numerical value of $\mu (t)$ in Eq. (31). As in the case of the stable fixed point $(v_0,w_0)$, the eigenvalues $\lambda _k(t)$, $k=\{1,2\}$, of $\varTheta _{ij}(t)$ characterize the distribution of random trajectories in the Poincaré section $\varSigma _{t}$ near a point $\big [\bar{v}(t),\bar{w}(t)\big ]$ of the stable limit cycle. The maximum of the largest eigenvalue indicates the SSF of the stable limit cycle. See Fig. 6b.

## Simulation results and discussion

In this section, numerical simulations are carried out with our model equation in the bistable regime to understand the ISR we observed in Fig. 2. We want to see how ISR depends on which basin of attraction the initial conditions are located in, and how the singular parameter $\varepsilon$ affects ISR. We provide a theoretical explanation of the numerical results in terms of the results obtained in the stochastic sensitivity analysis of our model equation. We recall that the differences in the SSFs and Mahalanobis distances of our stable attractors define the direction of noise-induced transitions between them.

**Fig. 7:**
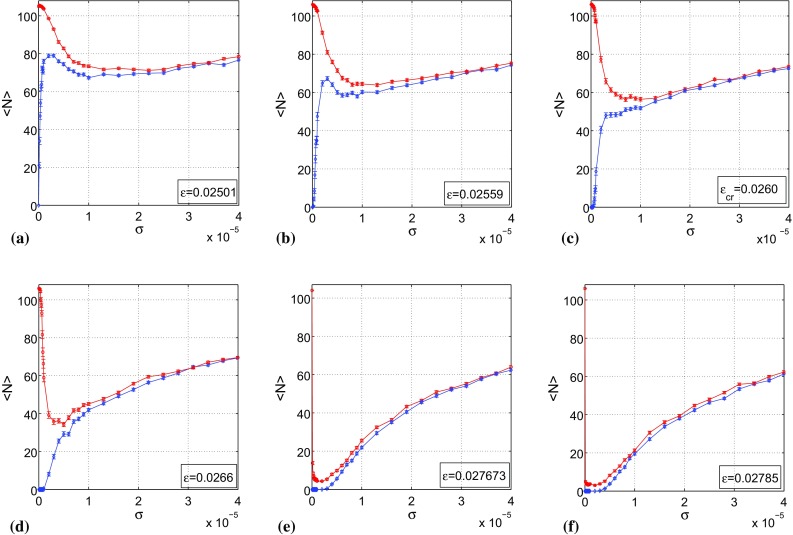
Mean number of spikes $\langle N\rangle$ versus noise amplitude $\sigma$ (200 trials for 7500 units of time interval each), for different singular parameter values $\varepsilon$ as indicated. ISR always occurs when $\big (v(0),w(0)\big )\in \mathcal {B}\big [\bar{v}(t),\bar{w}(t)\big ]$ (red curves). For $\big (v(0),w(0)\big )\in \mathcal {B}(v_0,w_0)$ (blue curves), ISR *only* occurs when $D_m(fp)<D_m(lc)$, i.e, when $\varepsilon \in (0.025,0.0260)$. $a=-\,0.05, b=1.0, c=2.0$. See text for details

Using the fourth-order Runge–Kutta algorithm for stochastic processes (Klasdin 1995), simulations are carried out for 200 realizations of the noise and for 7500 unit time intervals for each realization, a sufficiently long time interval for convergence of solutions for $0<\sigma \ll 1$. In Fig. 7, we depict the variations of the mean number of spikes $\langle N\rangle$ with the noise amplitude $\sigma$. The set of numerical results are for different values of Mahalanobis distances and SSFs of the stable attractors (encoded in the value of the singular parameter $\varepsilon$ as in Fig. 6).

Subthreshold responses are not counted as spikes. Again, we count a spike (supra-threshold response) when the action potential variable *v* is greater than or equal to the threshold value of $v_{th}=0.25$. We show simulation results for six values of the singular parameter $\varepsilon \in (\varepsilon _{hp},\varepsilon _{sn})$, namely: $\varepsilon =0.02501$ which is in the vicinity of the Hopf bifurcation value $\varepsilon _{hp}$ of the fixed point, $\varepsilon =0.02559$, $\varepsilon =0.0260$, $\varepsilon =0.0266$ at which both attractors have equal Mahalanobis distances, $\varepsilon =0.027673$ at which both attractors have equal SSFs, and $\varepsilon =0.02785$ which is in the vicinity of the saddle-node bifurcation value $\varepsilon _{sn}$ of the limit cycles.

The initial conditions $\big (v(0),w(0)\big )$ are fixed in every simulation. In Fig. 7, the red curves correspond to when $\big (v(0),w(0)\big )\in \mathcal {B}\big [\bar{v}(t),\bar{w}(t)\big ]$. In this case, when $\sigma =0$, there are 106 spikes. The inhibitory effect of the spiking activity begins as soon as $\sigma >0$, where we see the mean number of spikes $\langle N\rangle$ decreasing to a minimum value before increasing monotonically as $\sigma$ increases. We have ISR always occurring when $\big (v(0),w(0)\big )\in \mathcal {B}\big [\bar{v}(t),\bar{w}(t)\big ]$.

The blue curves correspond to the situation where $\big (v(0), w(0)\big )\in \mathcal {B}(v_0,w_0)$. In this case, when $\sigma =0$, we have no spike, $\langle N\rangle =0$, and as soon as $\sigma >0$, $\langle N\rangle$ only increases monotonically and ISR does not occur. However, in Fig. 7a, b (blue curves), interestingly, ISR does actually occur although $\big (v(0),w(0)\big )\in \mathcal {B}(v_0,w_0)$. We now explain these behaviors theoretically in terms of the Mahalanobis distances and the SSFs of the attractors.

In Fig. 7a (blue curve), $\varepsilon =0.02501\gtrapprox \varepsilon _{hp}$, in which case the Mahalanobis distance of the fixed point, $D_m(fp)$, is small and smaller than the Mahalanobis distance of the limit cycle, $D_m(lc)$. At this same value of $\varepsilon$, the SSF of the fixed point, $\lambda _{max}(fp)$, is high and far higher than the SSF of the limit cycle, $\lambda _{max}(lc)$. Therefore, with $\big (v(0),w(0)\big )\in \mathcal {B}(v_0,w_0)$, very weak-noise amplitudes are already capable of kicking the random trajectories out of $\mathcal {B}(v_0,w_0)$ into $\mathcal {B}\big [\bar{v}(t),\bar{w}(t)\big ]$, thereby increasing $\langle N\rangle$. For $0\le \sigma \le 0.25\times 10^{-5}$, $\langle N\rangle$ increases from 0 to a maximum of 79.8. This same interval of $\sigma$ is incapable of causing the reverse event, i.e., not strong enough to kick random trajectories back into $\mathcal {B}(v_0,w_0)$ because of a large $D_m(lc)$ and a low $\lambda _{max}(lc)$ and therefore, no inhibitory effect of the spiking activity. As a result, $\langle N\rangle$ can only increase monotonically for $0\le \sigma \le 0.25\times 10^{-5}$. As soon as $\sigma >0.25\times 10^{-5}$, it becomes strong enough to kick the random trajectories it previously kicked into $\mathcal {B}\big [\bar{v}(t),\bar{w}(t)\big ]$ back to $\mathcal {B}(v_0,w_0)$, thereby decreasing $\langle N\rangle$ (inhibiting the spiking activity) down to a minimum value of $\langle N\rangle =69.3$ at $\sigma =1.0\times 10^{-5}$, before increasing monotonically with $\sigma$.

From a series of simulations carried out for several different values of $\varepsilon \in (\varepsilon _{hp},\varepsilon _{cr}]$ (not all shown except for $\varepsilon =0.02501$, $\varepsilon =0.02559$, and $\varepsilon _{cr}=0.0260$ in Fig. 7a–c, respectively), ISR remarkably persisted with $\big (v(0),w(0)\big )\in \mathcal {B}(v_0,w_0)$, except at the critical value $\varepsilon _{cr}$, where it just disappeared. That is, as $\varepsilon$ increased in the interval $(\varepsilon _{hp},\varepsilon _{cr})$ (with increasing $D_{m}(fp)$ and residence time in $\mathcal {B}(v_0,w_0)$), ISR became less and less pronounced and eventually disappeared when $\varepsilon \ge \varepsilon _{cr}$. This is so because as $D_{m}(fp)$ becomes larger and larger with increasing $\varepsilon \ge \varepsilon _{cr}$, trajectories stay longer and longer in $\mathcal {B}(v_0,w_0)$ (and therefore there is no spike), and as $\sigma$ increases, it becomes at each time *just* strong enough to kick the trajectories into $\mathcal {B}\big [\bar{v}(t),\bar{w}(t)\big ]$ and hence $\langle N\rangle$ can only increase monotonically from $\langle N\rangle =0$ with $\sigma$. See the blue curves in Fig. 7c–f where $\varepsilon \ge \varepsilon _{cr}$, ISR does not occur as opposed to the cases in Fig. 7a, b (blue curves) where $\varepsilon \in (\varepsilon _{hp},\varepsilon _{cr})$.

Still in Fig. 7a (now the red curve), with $\big (v(0),w(0)\big )\in \mathcal {B}\big [\bar{v}(t),\bar{w}(t)\big ]$, in the thin interval of very weak-noise amplitudes $0\le \sigma <0.2\times 10^{-5}$, $\langle N\rangle$ is almost constant, near 106 (i.e., no considerable drop in $\langle N\rangle$ for this interval of $\sigma$). This “almost constant” value of $\langle N\rangle$ in that interval of $\sigma$ happens because at $\varepsilon =0.02501$, $D_{m}(lc)$ is large and $\lambda _{max}(lc)$ is very low and as $\big (v(0),w(0)\big )\in \mathcal {B}\big [\bar{v}(t),\bar{w}(t)\big ]$, trajectories have the tendency of staying in this basin of attraction for a very long time and therefore almost no inhibition of the spiking activity occurs for $0\le \sigma <0.2\times 10^{-5}$. As soon as $\sigma >0.2\times 10^{-5}$, it becomes strong enough to kick trajectories out of the relatively larger $\mathcal {B}\big [\bar{v}(t),\bar{w}(t)\big ]$. The inhibitory effect of noise then becomes pronounced with a clear decrease in $\langle N\rangle$ from about 106 to a minimum of 78.3 at $\sigma =2.24\times 10^{-5}$ before increasing monotonically with $\sigma$.

In Fig. 7d, $\varepsilon =0.0266$, $D_{m}(fp)=D_{m}(lc)$, with $\big (v(0),w(0)\big )\in \mathcal {B}\big [\bar{v}(t),\bar{w}(t)\big ]$ (red curve), there is a rapid decrease in $\langle N\rangle$ for weak $\sigma$. $\langle N\rangle$ moves from 106 to a minimum of 34.6 within $0\le \sigma \le 0.5\times 10^{-5}$ before increasing monotonically with increasing $\sigma$. A quicker decrease with a lower minimum in $\langle N\rangle$ as compared to the cases in Fig. 7a–c (red curves) occurs because of a smaller $D_{m}(lc)$ in Fig. 7d than in all previous cases, with therefore a shorter residence time in $\mathcal {B}\big [\bar{v}(t),\bar{w}(t)\big ]$.

Still in Fig. 7d with $\big (v(0),w(0)\big )\in \mathcal {B}(v_0,w_0)$ (blue curve), ISR disappears. Because at $\varepsilon =0.0266$ we have $D_{m}(fp)=D_{m}(lc)$, we explain this disappearance in terms of the other factor determining the residence time in basins of attraction, i.e., the SSFs of the attractors. At $\varepsilon =0.0266$, $\lambda _{max}(fp)$ is still sufficiently high (with $\lambda _{max}(lc)<\lambda _{max}(fp)$, which means that the fixed point is more sensitive to noise than the limit cycle) and therefore, even weak-noise amplitudes have the tendency of kicking the trajectories initially in $\mathcal {B}(v_0,w_0)$ into $\mathcal {B}\big [\bar{v}(t),\bar{w}(t)\big ]$ (thereby increasing $\langle N\rangle$). As in the cases of Fig. 7a, b (blue curves), one will expect that $\langle N\rangle$ increases with $\sigma \ge 0$ up to a certain maximum, and then start to decrease through the inhibitory effect of noise. This is not happening in Fig. 7d (and also already in Fig. 7c blue curve) firstly because $D_{m}(lc)$ is still sufficiently large (even though equal to $D_{m}(fp)$) to keep the trajectories in $\mathcal {B}\big [\bar{v}(t),\bar{w}(t)\big ]$. Secondly, and mainly because $\lambda _{max}(lc)<\lambda _{max}(fp)$ at $\varepsilon =0.0266$, which means that when trajectories get into $\mathcal {B}\big [\bar{v}(t),\bar{w}(t)\big ]$, they prefer to stay in this basin. And of course stronger and stronger noise only increases $\langle N\rangle$.

In Fig. 7e, $\varepsilon =0.027673$, $D_{m}(lc)$ is much smaller and $\lambda _{max}(lc)$ is much higher than in the previous cases, but $\lambda _{max}(fp)=\lambda _{max}(lc)$. For the case $\big (v(0),w(0)\big )\in \mathcal {B}\big [\bar{v}(t),\bar{w}(t)\big ]$ (red curve), we therefore have a faster drop in $\langle N\rangle$, i.e., from 106 to a minimum of 6.4 within $0\le \sigma \le 0.25\times 10^{-5}$. With $\big (v(0),w(0)\big )\in \mathcal {B}(v_0,w_0)$ (blue curve) ISR disappears for basically the same reason as previously given. In this case, for $0\le \sigma \le 0.25\times 10^{-5}$, $\langle N\rangle$ remains at zero (since $D_m(fp)>D_m(lc)$) and as $\sigma$ increases and becomes stronger, random trajectories start to jump into $\mathcal {B}\big [\bar{v}(t),\bar{w}(t)\big ]$ (thus increasing $\langle N\rangle$) and remain in this basin for stronger and stronger noise with the immediate consequence of just increasing $\langle N\rangle$.

In Fig. 7f, $\varepsilon =0.02785\lessapprox \varepsilon _{sn}$, $D_{m}(lc)$ is the smallest and $\lambda _{max}(lc)$ very high. For $\big (v(0),w(0)\big )\in \mathcal {B}\big [\bar{v}(t),\bar{w}(t)\big ]$ (red curve), there is a much more rapid and deeper drop in $\langle N\rangle$ with weak-noise amplitudes as compared to all the previous cases with a well defined minimum value of $\langle N\rangle =4.1$ at $\sigma =0.25\times 10^{-5}$ and then a monotonic increase in $\langle N\rangle$ with increasing $\sigma$. In this case, for some noise realizations, the number of spikes could drop down to zero. That is, weak-noise amplitudes completely terminate the spiking dynamics.

For $\big (v(0),w(0)\big )\in \mathcal {B}(v_0,w_0)$ (blue curve), because $D_{m}(fp)$ is larger at $\varepsilon =0.02785$, the residence time in $\mathcal {B}(v_0,w_0)$ is on average the longest for weak-noise amplitudes compared to all previous cases. We have $\langle N\rangle =0$ for $0\le \sigma <0.30\times 10^{-5}$. For $\sigma \ge 0.30\times 10^{-5}$, the noise is now sufficiently strong to start kicking trajectories into $\mathcal {B}\big [\bar{v}(t),\bar{w}(t)\big ]$ causing an increase in $\langle N\rangle$. And as $\sigma$ becomes stronger, it keeps driving the neuron and so the trajectories remain $\mathcal {B}\big [\bar{v}(t),\bar{w}(t)\big ]$ with $\langle N\rangle$ increasing monotonically with $\sigma$.

## Conclusion

The effects of weak-noise amplitudes on the spiking dynamics of the FHN neuron model were investigated. Through bifurcation and slow-fast analyses, we determined the conditions on the parameter space for the establishment of a bi-stability regime consisting of a unique stable fixed point and a stable unforced limit cycle. This bi-stability regime induces a sensitivity to initial conditions in the immediate neighborhood of the separatrix isolating the basins of attraction of the attractors. Introducing noise to the system then causes transitions from the basin of attraction of the fixed point to that of the limit cycle (the neuron gets into the spiking state) and as well, from the basin of attraction of the limit cycle to that of the fixed point (the neuron gets into the quiescent state, no spiking).

We observed that in this bistable regime, weak-noise amplitudes may decrease the mean number of spikes down to a minimum value after which it increases monotonically as the noise strength increases. We showed that this phenomenon always occurred if the initial conditions were chosen from the basin attraction of the stable limit cycle. For initial conditions in the basin of attraction of the stable fixed point, the phenomenon disappeared, unless the timescale separation parameter $\varepsilon$ of the neuron model is bounded between $\varepsilon _{hp}=0.0250$, its Hopf bifurcation value and $\varepsilon _{cr}=0.0260$. Furthermore, the phenomenon became less and less pronounced as $\varepsilon$ increased in the interval $(\varepsilon _{hp},\varepsilon _{cr})$ and disappeared at $\varepsilon \ge \varepsilon _{cr}$.

We have seen that the stochastic sensitivity functions of the stable attractors and their Mahalanobis distances from the separatrix, which both determine the length of the residence time of random trajectories in each state (quiescent or spiking state of the neuron), themselves depended on the timescale separation parameter $\varepsilon$ of the model. From this dependence, we provided a theoretical explanation of the noise-induced phenomenon of ISR in terms of the stochastic sensitivity functions and the Mahalanobis distances of the stable attractors.

Finally, we see that the key to ISR is the multi-stability between fixed points and limit cycles, a characteristic of dynamical systems with subcritical Hopf bifurcations. To obtain bi-stability, in the present work, a careful relative positioning of the fixed point on the critical manifold was made. We can see in (Yamakou and Jost 2018) how a change in the relative position of the fixed and fold points on the critical manifold brings about a completely different dynamical behavior in the same weak-noise limit. Plausible implications of ISR in information processing and transmission in neurons are discussed in (Buchin et al. 2016).

## Figures and Tables

**Fig. 8:**
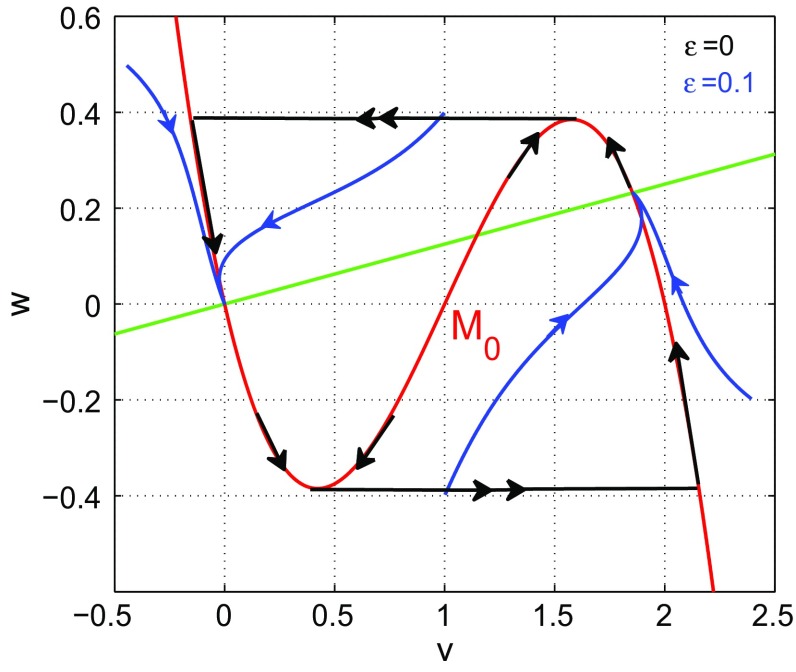
All the black trajectories (with single and double arrows) represent the solution of the slow flow of Eq. (A.31) (with $\varepsilon =0$) on the critical manifold ${\mathcal {M}}_0$ (the red curve). The black trajectories with single arrow evolve on ${\mathcal {M}}_0$ toward the singular points at $v_\pm$ located at the extrema of ${\mathcal {M}}_0$, and horizontally leave ${\mathcal {M}}_0$ at $v_\pm$ as shown by the black horizontal trajectories with double arrows. The trajectories of the flow of Eq. (2) (with $\varepsilon =0.1$) on the slow manifold ${\mathcal {M}}_{\varepsilon }$ (not shown but at a distance $\mathcal {O}(\varepsilon )$ from ${\mathcal {M}}_0$) converge to the stable fixed points $v_0=0$ and $v_2$ located at the intersection of the *w*-nullcline (the green line) and the decreasing parts of ${\mathcal {M}}_{0}$, without a limit cycle occurring. $a=2.0, b=1.0, c=8.0, \sigma =0.0$

## Appendix A: Complete bifurcation analysis

We now consider the deterministic dynamics corresponding to Eq. (2) when $\sigma = 0$ and perform an explicit bifurcation and slow-fast analysis through which we find the parametric conditions for the establishment of a bi-stability regime consisting of a stable fixed point and a stable unforced limit cycle. We note again that our model has no deterministic input current. At the fixed points $(v_e,w_e)$ (rest states of the neuron), the variables *v*(*t*) and *w*(*t*) reach a stationary state, while the set of fixed points is defined by the intersection of nullclines as

A.1 $$\begin{aligned} (v_e,w_e):=\Big \{(v,w)\in {\mathbb {R}}^2:f(v,w)=g(v,w)=0\Big \}. \end{aligned}$$

From Eq. (A.1), we obtain the fixed point equations

A.2 $$\begin{aligned} {\begin{matrix} \left\{ \begin{array}{lcl} \frac{b}{c}v&{}=&{}-v^3+(a+1)v^2-av,\\ w&{}=&{}\frac{b}{c}v, \end{array}\right. \end{matrix}} \end{aligned}$$

which has the solutions for *v* as

A.3 $$\begin{aligned} v_0=0 \text { and } v_{1,2}=\frac{a+1}{2}\pm \sqrt{\frac{(a-1)^2}{4} -\frac{b}{c}}, \end{aligned}$$

where the solutions $v_{1,2}$ exist if

A.4 $$\begin{aligned} \frac{(a-1)^2}{4}\ge \frac{b}{c}. \end{aligned}$$

We always assume $b,c >0$. When $0<a<1$, we then have

A.5 $$\begin{aligned} v_0<a< v_{1,2} <1, \end{aligned}$$

but when $a<0$, this no longer holds.

A bifurcation occurs when some of these fixed points coincide. We have $v_1=v_2$ if and only if

A.6 $$\begin{aligned} \frac{(a-1)^2}{4} =\frac{b}{c}, \end{aligned}$$

and we have $v_0=v_1$ if

A.7 $$\begin{aligned} \frac{b}{c}=-\,a. \end{aligned}$$

When $a=-\,1$ and $\frac{b}{c}=1$, all three fixed points coincide. The case $a=-\,1$ is somewhat simpler because the cubic polynomial in Eq. (A.2) reduces to

A.8 $$\begin{aligned} -\,v^3+v=\frac{b}{c}v, \end{aligned}$$

and much of the subsequent analysis will only be carried out for that particular case. For the moment, however, we return to the general case. The linearization of Eq. (2) at such a fixed point $v_\star$ is

A.9 $$\begin{aligned} {\begin{matrix} \left\{ \begin{array}{lcl} \varepsilon \frac{\hbox {d}\varphi }{\hbox {d}\tau }&{}=&{} -3v_\star ^2\varphi +2(a+1)v_\star \varphi -a\varphi -\eta ,\\ \\ \frac{\hbox {d}\eta }{\hbox {d}\tau }&{}=&{} b\varphi -\,c \eta . \end{array}\right. \end{matrix}} \end{aligned}$$

In order to determine the bifurcation behavior at such a fixed point, we need to investigate the eigenvalues of the Jacobian matrix given by

A.10 $$\begin{aligned} J_{ij}=\left( \begin{array}{cc} \frac{1}{\varepsilon }(-\,3v_\star ^2 +2(a+1)v_\star -a )&{}\,\,\,\,\, -\,\frac{1}{\varepsilon }\\ \\ b&{}\,\,\,\,\, -\,c \end{array} \right) . \end{aligned}$$

The stability of the fixed points $v_\star$ will depend on the signs of the trace and determinant of $J_{ij}$. For a fixed point $v_\star$ to be stable, we should have $\mathrm {tr}J_{ij}<0$ and $\mathrm {det} J_{ij}>0$. Since $\varepsilon ,c >0$, we have $\mathrm {tr}J_{ij}<0$ and $\mathrm {det} J_{ij}>0$ only if

A.11 $$\begin{aligned} -3v_\star ^2 +2(a+1)v_\star -a<0. \end{aligned}$$

Eq. (A.11) means that the fixed point $v_\star$ has to be on the part of the cubic polynomial $-v^3+(a+1)v^2-av$ that has a negative derivative (see the red curve in Fig. 3b). When we have three distinct fixed points $v_\star$ in Eq. (A.3), this could hold for the leftmost and the rightmost of them, and these two would then be stable, while the middle one would be unstable. The fixed point $v_0=0$ is stable for $a>0$, and unstable for $a<0$. We should point out, however, that Eq. (A.11) is a sufficient, but not a necessary condition for the stability of a fixed point.

When we vary the parameters so that two of the fixed points merge, we obtain a saddle-node type bifurcation. In the case $a=-\,1$, the fixed points $v_{1,2}$ (which are symmetric in this case, that is, $v_1=-\,v_2$) are stable according to Eq. (A.11) if and only if

A.12 $$\begin{aligned} v_1 < -\frac{1}{\sqrt{3}}, \end{aligned}$$

that is, if $v_1$ is to the left of the local minimum of the cubic polynomial $w=-\,v^3+v$ (equivalently, $v_2>\frac{1}{\sqrt{3}}$ is to the right of the local maximum). The limiting case

A.13 $$\begin{aligned} v_1 =-\,\frac{1}{\sqrt{3}}, \end{aligned}$$

of Eq. (A.12) corresponds to $\frac{b}{c}=\frac{2}{3}$, by Eq. (A.3). In this case, $v_1$ is precisely the minimum of the cubic polynomial $-v^3 +v$ in Eq. (A.8). Likewise, $v_2$ then is the maximum of that polynomial. This case will reoccur below in Eq. (A.26).

When Eq. (A.4) is not satisfied, $v_0=0$ is the only fixed point. For $v_0$, the determinant of Eq. (A.10) is $\frac{1}{\varepsilon }(ac+b)$, and the trace is $-\frac{a}{\varepsilon }-c$. Thus, $v_0$ is stable when *a* is not too negative, but in the limit $\varepsilon \rightarrow 0$, stability only persists for $a\ge 0$. (Recall here that Eq. (A.11) was sufficient, but not necessary for the stability of a fixed point $v_\star$.)

We obtain complex conjugate eigenvalues if $0>\frac{1}{4}(\mathrm {tr} J_{ij} )^2 -\mathrm {det} J_{ij}$, and the real part of these eigenvalues vanishes when $\mathrm {tr} J_{ij}=0$. For Eq. (A.10), we thus have the condition for complex conjugate eigenvalues as

A.14 $$\begin{aligned} {\begin{matrix} \left\{ \begin{array}{lcl} \frac{1}{\varepsilon }(-\,3v_\star ^2 +2(a+1)v_\star -a) +c -2\sqrt{\frac{b}{\varepsilon }}<0,\\ \frac{1}{\varepsilon }(-\,3v_\star ^2 +2(a+1)v_\star -a) +c +2\sqrt{\frac{b}{\varepsilon }}>0, \end{array}\right. \end{matrix}} \end{aligned}$$

and the condition for a vanishing real part

A.15 $$\begin{aligned} \frac{1}{\varepsilon }(-\,3v_\star ^2 +2(a+1)v_\star -a)-c=0. \end{aligned}$$

In order to be able to satisfy Eq. (A.14) and Eq. (A.15) simultaneously (and therefore have a Hopf bifurcation), the coefficients $b,c\ (>0)$ need to satisfy

A.16 $$\begin{aligned} c^2 <\frac{b}{\varepsilon }, \end{aligned}$$

which is easily satisfied for small $\varepsilon >0$, since we assume $b>0$.

When we solve Eq. (A.15) for $v_\star$, we obtain

A.17 $$\begin{aligned} v_{\star \star }=\frac{a+1}{3}\pm \sqrt{\frac{(a+1)^2}{9}-\frac{a+\varepsilon c}{3}}, \end{aligned}$$

which we can solve as long as

A.18 $$\begin{aligned} 3\varepsilon c \le a^2-a +1. \end{aligned}$$

We can then check when a solution $v_{\star \star }$ of Eq. (A.17) coincides with one of the points given by Eq. (A.3), in order to get a Hopf bifurcation at one of those fixed points. We had already observed above that a fixed point on the decreasing part of the cubic *v*-nullcline curve $w=-\,v^3+(a+1)v^2-av$ (the red curve in Fig. 3b) is stable as the eigenvalues of the linearization then have negative real parts. But this was a sufficient, but not a necessary condition. Therefore, when we vary the slope $\frac{b}{c}$ of the *w*-nullcline and move a fixed point to the middle increasing part of the cubic polynomial, that fixed point may eventually lose its stability. When the parameter regime just identified for a Hopf bifurcation is right, that loss of stability will occur through a Hopf bifurcation.

In order to see the significance of Eq. (A.18), we observe that the local extrema of the cubic polynomial $-v^3+(a+1)v^2-av$ are at

A.19 $$\begin{aligned} v_\pm =\frac{a+1}{3}\pm \sqrt{\frac{(a+1)^2}{9}-\frac{a}{3}}, \end{aligned}$$

and since $\varepsilon c >0$, $\frac{a+1}{3}- \sqrt{\frac{(a+1)^2}{9}-\frac{a+\varepsilon c}{3}}> \frac{a+1}{3}- \sqrt{\frac{(a+1)^2}{9}-\frac{a}{3}}$, that is, the left Hopf bifurcation is to the right of the minimum of our cubic polynomial, hence on its ascending branch. Whenever a fixed point is on the left descending branch, that is, to the left of that minimum, it is stable, and stability persists a little into the ascending branch, but when $\varepsilon \rightarrow 0$, the Hopf bifurcation point, that is, where the fixed point looses its stability, moves toward that minimum. For $\varepsilon >0$, the Hopf bifurcation occurs to the right of the minimum.

We return to the investigation of Eq. (A.15). In particular, for $$v_\star =0$$, Eq. (A.15) does not have a solution under our constraint $$ c>0$$ when also $$a>0$$. When $$a<0$$, Eq. (A.15) is satisfied for $$v_\star =0$$ when

A.20 $$\begin{aligned} a=-\,\varepsilon c. \end{aligned}$$

When $a>0$ and Eq. (A.18) holds, the solutions $v_{\star \star }$ of Eq. (A.17) are positive, and we might then tune the parameter *b* which does not occur in Eq. (A.17) so that one of those $v_{\star \star }$ coincides with $v_1$ or $v_2$ from Eq. (A.3).

When we have equality in Eq. (A.16), i.e., $c^2 =\frac{b}{\varepsilon }$, Eq. (A.20) becomes

A.21 $$\begin{aligned} a=-\,\frac{b}{c}, \end{aligned}$$

that is, Eq. (A.7). In that case, we have a limit of a Hopf bifurcation co-occurring with a saddle-node bifurcation.

For $v_\star =v_{1,2}$ in Eq. (A.3), we obtain

A.22 $$\begin{aligned}&2(a+1)\left( \frac{a+1}{2}\pm \sqrt{\frac{(a-1)^2}{4} -\frac{b}{c}}\right) -a\nonumber \\&\quad =3\left( \frac{a+1}{2}\pm \sqrt{\frac{(a-1)^2}{4} -\frac{b}{c}}\right) ^2+\varepsilon c, \end{aligned}$$

that is,

$$\begin{aligned} -\frac{a^2}{2} +a -\frac{1}{2} +3\frac{b}{c}-\varepsilon c=\pm (a+1)\sqrt{\frac{(a-1)^2}{4}-\frac{b}{c}}, \end{aligned}$$

which implies

$$\begin{aligned}&\frac{1}{4}(a-1)^4 +(3\frac{b}{c} -\varepsilon c)^2 -(a-1)^2(3\frac{b}{c} -\varepsilon c)\\&\quad =\frac{1}{4}(a-1)^2(a+1)^2 -(a+1)^2\frac{b}{c}, \end{aligned}$$

hence

$$\begin{aligned}&-a^3+2a^2-a -2a^2\frac{b}{c}+8a\frac{b}{c}-2\frac{b}{c} +a^2 \varepsilon c \\&\quad -2a\varepsilon c +\varepsilon c +9\frac{b^2}{c^2} -6b\varepsilon +\varepsilon ^2 c^2=0, \end{aligned}$$

which is equivalent to

A.23 $$\begin{aligned}&(a-1)^2(-\,a -2\frac{b}{c})+4a\frac{b}{c} +9\frac{b^2}{c^2}+\varepsilon ^2 c^2\nonumber \\&\quad =\varepsilon (-\,(a-1)^2 c +6b). \end{aligned}$$

Thus, Eq. (A.23) is the condition on the parameters for a Hopf bifurcation at one of the equilibria $v_{1,2}$.

In particular, in the limit $\varepsilon =0$, we get the condition

A.24 $$\begin{aligned} (a-1)^2(a +2\frac{b}{c})-4a\frac{b}{c} =9\frac{b^2}{c^2}. \end{aligned}$$

For the case $a=-\,1$, we directly get from Eq. (A.22)

A.25 $$\begin{aligned} 3\frac{b}{c}-2=\varepsilon c. \end{aligned}$$

Thus, for $\varepsilon >0$, in Eq. (A.25), we have $\frac{b}{c} >\frac{2}{3}$, and therefore the fixed point is to the right of the minimum, that is, on the increasing part of the cubic curve $-v^3+v$, in accord with what we had said after Eq. (A.17).

In the limit $\varepsilon =0$, we obtain

A.26 $$\begin{aligned} \frac{b}{c}=\frac{2}{3}, \end{aligned}$$

as the condition for a Hopf bifurcation at an equilibrium $v_{1,2}$. This is the case of Eq. (A.13) (recalling Eq. (A.3)). Here, $v_1$ and $v_2$ lose their stability, and below in the slow-fast analysis, these are also the points where the critical manifold will not be normally hyperbolic, and where a switch from slow to fast dynamics will occur, generating an unforced limit cycle. Since we had identified Eq. (A.26) as the parameter constellation for a Hopf bifurcation, this is a singular limiting situation for a Hopf bifurcation.

## Appendix B: Slow-fast analysis

Here we combine slow-fast analysis and the results from the bifurcation analysis above to understand how a stable unforced limit cycle emerges from Eq. (2) (with $\sigma =0$) and coexist with a unique stable fixed point. For a more detailed introduction to multiple timescale dynamics see (Kuehn 2015). In the singular limit $\varepsilon =0$, we define the critical manifold ${\mathcal {M}}_0$ of Eq. (2) which coincides with our cubic polynomial nullcline:

A.27 $$\begin{aligned} {\mathcal {M}}_0:=\Big \{(v,w)\in {\mathbb {R}}^2:f(v,w)=0\Big \}. \end{aligned}$$

${\mathcal {M}}_0$ can be viewed as the algebraic constraint of the differential-algebraic slow subsystem Eq. (A.31) whose initial conditions must satisfy this constraint for solutions to exist. From Eq. (A.27), we have

A.28 $$\begin{aligned} \frac{\hbox {d}w}{\hbox {d}\tau }= -3v^2 +2(a+1)v-a, \end{aligned}$$

with

A.29 $$\begin{aligned} \frac{\hbox {d}w}{\hbox {d}\tau }{\left\{ \begin{array}{ll}<0,\,\, \text {for} {\left\{ \begin{array}{ll}v<\frac{a+1}{3}-\frac{1}{3}\sqrt{a^2-a +1} \\ v> \frac{a+1}{3} +\frac{1}{3}\sqrt{a^2-a +1} \end{array}\right. }\\ \\ =0,\,\,\text {for}\,\,v=\frac{a+1}{3} \pm \frac{1}{3}\sqrt{a^2-a +1} \\ \\>0,\,\, \text {for} {\left\{ \begin{array}{ll} v>\frac{a+1}{3} -\frac{1}{3}\sqrt{a^2-a +1}\\ v< \frac{a+1}{3} +\frac{1}{3}\sqrt{a^2-a +1} \end{array}\right. } \end{array}\right. } \end{aligned}$$

Thus, ${\mathcal {M}}_0$ naturally splits into three parts: two decreasing stable branches and a middle increasing unstable branch, see the red curve in Figs. 8 or 3b. ${\mathcal {M}}_0$ looses its normal hyperbolicity at the two singular points $v_\pm = \frac{a+1}{3} \pm \frac{1}{3}\sqrt{a^2-a+1}$, where it changes its stability property. These two points are the local extrema of ${\mathcal {M}}_0$. In fact, at $v_\pm = \frac{a+1}{3} \pm \frac{1}{3}\sqrt{a^2-a+1}$, the existence and uniqueness theorems for ordinary differential equations (ODEs) do not longer apply, and because of this, the solutions of the slow subsystem in Eq. (A.31) are forced to leave ${\mathcal {M}}_0$ at these singular points.

In the singular limit $\varepsilon =0$, the dynamics of the fast variable *v* on ${\mathcal {M}}_0$ (i.e., a 1-D dynamical system of the variable *v* whose phase space is ${\mathcal {M}}_0$), is obtained from Eq. (A.27) by implicit differentiation

A.30 $$\begin{aligned} (-\,3v^2 +2(a+1)v-a)\frac{dv}{d\tau }=\frac{\hbox {d}w}{\hbox {d}\tau }=bv-cw, \end{aligned}$$

and using the algebraic constraint on *w* in Eq. (A.27), we eliminate this variable to get the slow flow for the variable *v* as

A.31 $$\begin{aligned} \frac{dv}{d\tau }=\frac{bv -c (-\,v^3+(a+1)v^2-av)}{-3v^2 +2(a+1)v-a}\ , \end{aligned}$$

which, of course, becomes singular at the points $v_\pm$. In fact, at these points, since the critical manifold ${\mathcal {M}}_0$ loses its stability, the slow flow in Eq. (A.31) should detach from ${\mathcal {M}}_0$ and should become fast, that is, satisfy $\frac{\hbox {d}w}{\hbox {d}\tau }=0$ and horizontally jump to another stable branch of ${\mathcal {M}}_0$.

In Fig. 8, all the black trajectories (with single and double arrows) represent the solution of the slow flow of Eq. (A.31) on ${\mathcal {M}}_0$. Because of the failure of the existence and uniqueness theorems of ODEs at $v_-$, the lower horizontal (fast) trajectory (in black with double arrow) leaves the left stable part of ${\mathcal {M}}_0$ at its local minimum at $v_-$ to the right stable part of ${\mathcal {M}}_0$. For the same reason, the upper horizontal (fast) trajectory (in black with double arrow) leaves the right stable part of ${\mathcal {M}}_0$ at its local maximum at $v_+$ to the left stable part of ${\mathcal {M}}_0$. In this same figure, the non-horizontal (slow) trajectories (all in black with a single arrow) of Eq. (A.31), evolve on ${\mathcal {M}}_0$ toward the singular points at $v_\pm$, where they eventually leave ${\mathcal {M}}_0$.

The solution (or more precisely, the singular solution with $\varepsilon =0$) of the slow subsystem in Eq. (A.31) is related to the solution of the full system Eq. (2) with $\varepsilon >0$ by Fenichel’s theorem (Fenichel 1971, 1979). By that theorem, for $0<\varepsilon \ll 1$, the slow manifold ${\mathcal {M}}_{\varepsilon }$ is a perturbation of the critical manifold ${\mathcal {M}}_0$, and it has the following properties:

- (F1) ${\mathcal {M}}_{\varepsilon }$ is diffeomorphic to ${\mathcal {M}}_0$.
- (F2) ${\mathcal {M}}_{\varepsilon }$ has distance $\mathcal {O}(\varepsilon )$ from ${\mathcal {M}}_0$.
- (F3) The flow on ${\mathcal {M}}_{\varepsilon }$ converges to the slow flow on ${\mathcal {M}}_0$ as $\varepsilon \rightarrow 0$.
- (F4) ${\mathcal {M}}_{\varepsilon }$ is $C^r$-smooth for any $r<\infty$ (as long as $f,g\in C^\infty$).
- (F5) ${\mathcal {M}}_{\varepsilon }$ is normally hyperbolic, with the same stability properties w.r.t. the fast variable *v* as ${\mathcal {M}}_0$ (attracting, repelling or saddle-type).
- (F6) ${\mathcal {M}}_{\varepsilon }$ is usually not unique. Manifolds satisfying (F1)-(F5) lie at distance $\mathcal {O}\big (\exp (K /\varepsilon )\big )$ from each other $K>0$, $K=\mathcal {O}(1)$.
- (F7) Similar conclusions hold for the stable/unstable manifolds of ${\mathcal {M}}_0$.

Most importantly, the flow of Eq. (2) (with $0<\varepsilon \ll 1$) on ${\mathcal {M}}_{\varepsilon }$ will follow the slow flow of Eq. (A.31) (with $\varepsilon =0$) on ${\mathcal {M}}_0$. By the definitions in Eq. (A.1) and Eq. (A.27), we see that the fixed points $v_\star$ in Eq. (A.3) also lie on ${\mathcal {M}}_0$. We have the fixed points $v_0=0$ and $v_2$ on the decreasing part of ${\mathcal {M}}_0$, and they are therefore stable, while the fixed point $v_1$ is located between $v_0=0$ and $v_2$ and unstable.

In Fig. 8, the blue trajectories with arrows pointing in the direction of the flow on the slow manifold ${\mathcal {M}}_{\varepsilon }$ (not shown, but at a distance $\mathcal {O}(\varepsilon )$ from ${\mathcal {M}}_0$) represent solutions of Eq. (2) with $\varepsilon =0.1$. They converge toward to the stable fixed points $v_0$ and $v_2$ located, respectively, on the left and right stable decreasing parts of the slow manifold ${\mathcal {M}}_{\varepsilon }$.

For a better visualization, we can henceforth discuss the dynamics with respect to ${\mathcal {M}}_0$, since Fenichel’s theorem tells us that the same dynamical behavior takes place on ${\mathcal {M}}_{\varepsilon }$. With the fixed point configuration: $v_0<v_1<v_2$ with $v_0$ and $v_2$ stable and located, respectively, on the left and right decreasing parts of ${\mathcal {M}}_0$, $v_1$ unstable and located on the increasing part of ${\mathcal {M}}_0$, trajectories of Eq. (2) cannot exhibit a spiking behavior (i.e., a limit cycle solution cannot emerge). This is because trajectories converge and stay at the stable fixed points $v_0$ and $v_2$ whenever they encounter them on decreasing parts of ${\mathcal {M}}_{0}$. Hence, these trajectories cannot evolve and reach the singular points $v_{\pm }$ located at the extrema of ${\mathcal {M}}_{0}$, where they can jump from the left to the right and then from the right to the left part of ${\mathcal {M}}_{0}$ to produce a limit cycle solution. In the blue trajectories in Fig. 8, they stick at the fixed points $v_0=0$ and $v_2$.

Thus, it depends on the relative positions of the singular points at $v_\pm$ and the fixed points at $v_0$ and $v_2$ on the critical manifold ${\mathcal {M}}_{0}$ whether the flow of Eq. (2) will first reach a stable fixed point (and stay there with no possibility for a limit cycle) or first reach a singular point $v_{\pm }$ and detach from ${\mathcal {M}}_{0}$ with the emergence of a limit cycle.

When, say, $v_0$ is the smallest fixed point and is located to the right of $v_-$ and $v_2$, the largest fixed point, to the left of $v_+$, we expect a periodic cycle for $0<\varepsilon \ll 1$. For instance, when we start close to the left stable branch of ${\mathcal {M}}_0$, by Fenichel’s theorem, the flow closely and slowly follows ${\mathcal {M}}_0$ until we get into the vicinity of $v_-$. There, ${\mathcal {M}}_0$ becomes unstable, and the flow will move away from it and become fast and therefore move not perfectly horizontally this time, but with some inclination (because $\varepsilon >0$) to the right until it comes into the vicinity of the right stable branch of ${\mathcal {M}}_0$. It will then again move slowly and close to ${\mathcal {M}}_0$ until it gets into the vicinity of $v_+$. Hence it moves away fast, also not perfectly horizontally but with some inclination to the left, until it encounters the left stable branch again, and the cycle repeats.

We therefore see that even in the absence of a deterministic input current, the slow-fast structure of Eq. (2) (with $\sigma =0$) can naturally induce a limit cycle solution (spiking) if the flow in Eq. (2) leaves from the neighborhood of the stable parts of ${\mathcal {M}}_0$ at the singular points $v_{\pm }$ before it encounters a stable fixed point. This can only happen if the fixed point is located to right of $v_-$ or to the left of $v_+$.

Comparing Eq. (A.11) and Eq. (A.28), we see that the fixed point $v_\star$ is stable for all $\varepsilon >0$ if it is to the left of the minimum of ${\mathcal {M}}_0$. In that case, it is on the left stable branch of ${\mathcal {M}}_0$, and the dynamics on that stable branch will therefore converge toward $v_\star$, without a limit cycle emerging. Analogously, a fixed point $v_\star$ is stable if it is on the right stable branch of ${\mathcal {M}}_0$ with no possibility for a limit cycle as well. Again, however, these are sufficient, but not necessary conditions for stability of the fixed points. For $\varepsilon >0$, stability persists a little into the middle (increasing) part of ${\mathcal {M}}_0$ as we have found when we discussed the possibility of a Hopf bifurcation. Therefore, to have a bi-stability regime consisting of a stable fixed point and a stable unforced limit cycle, the fixed point should be located on the middle unstable branch of ${\mathcal {M}}_0$ and has be to stable. This can be done by choosing the parameters such that the fixed point is located between the minimum $v_-$ of ${\mathcal {M}}_0$ and its Hopf bifurcation value.

For example, in the case where $v_0=0$ is the only fixed point, that is we choose *a*, *b*, and *c* such that Eq. (A.4) is not satisfied. The persistence of stability on the middle unstable part of ${\mathcal {M}}_0$ also occurs for $v_0=0$. For $a<0$, $v_0=0$ is to the right of the minimum $v_-=\frac{a+1}{3} - \sqrt{\frac{(a+1)^2}{9} -\frac{a}{3}}$ of ${\mathcal {M}}_0$ (i.e., $v_-<v_0$) and therefore in the region where it eventually loses its stability through an Hopf bifurcation when $\varepsilon$ increases. It now suffices to choose specific values of *a* ($a<0$) and *c* (both values of *a* and *c* also not satisfying Eq. (A.4)) such that $v_0<-\frac{a}{c}=\varepsilon$ (see Eq. (A.20)) to have a *stable* fixed point $v_0$ to the right of $v_-$.

With the stable fixed point $v_0=0$ on the middle part of ${\mathcal {M}}_0$, and from the slow-fast analysis above, we also have a stable limit cycle surrounding this fixed point. For topological reasons these attractors should be separated from each other by a repeller, in this case an unstable limit cycle. In fact, the unstable limit cycle is the boundary of the basin of attraction of the stable fixed point $v_0$. This indicates that the Hopf bifurcation of the fixed point $v_0$ which eventually occurs as $\varepsilon$ increases should be subcritical. Which of the stable attractors attracts a trajectory then depends on whether the initial conditions are located in the region bounded by the unstable limit cycle or out of this region.

## References

[CR1] Bashkirtseva I, Perevalova TV (2007). Analysis of stochastic attractors under the stationary point-cycle bifurcation. Autom Remote Control.

[CR2] Bashkirtseva I, Ryashko L (2004). Stochastic sensitivity of 3D-cycles. Math Comput Simul.

[CR3] Bashkirtseva I, Ryashko L, Slepukhina E (2014). Noise-induced oscillating bistability and transition to chaos in Fitzhugh-Nagumo model. Fluct Noise Lett.

[CR4] Bertram TVR, Tabak J, Wechselberger M (2010). Mixed mode oscillations as a mechanism for pseudo-plateau bursting. J Comput Neurosci.

[CR5] Buchin A, Rieubland S, Häusser M, Gutkin BS, Roth A (2016). Inverse stochastic resonance in cerebellar Purkinje cells. PLoS Comput Biol.

[CR6] Collins JJ, Carson CC, Imhoff TT (1995). Aperiodic stochastic resonance in excitable systems. Phys Rev E.

[CR7] Cymbalyuk G, Shilnikov A (2005). Coexistence of tonic spiking oscillations in a leech neuron model. J Comput Neurosci.

[CR8] Durrett R (1996). Probability: theory and examples.

[CR9] Fenichel N (1971). Persistence and smoothness of invariant manifolds for flows. Indiana Univ Math J.

[CR10] Fenichel N (1979). Geometric singular perturbation theory for ordinary differential equations. J Differ Equ.

[CR11] FitzHugh R (1961). Impulses and physiological states in theoretical models of nerve membrane. Biophys J.

[CR12] Freidlin MI, Wentzell AD (1998). Random perturbations of dynamical systems.

[CR13] Gutkin BS, Jost J, Tuckwell HC (2008). Transient termination of spiking by noise in coupled neurons. Europhys Lett.

[CR14] Gutkin BS, Jost J, Tuckwell HC (2009). Inhibition of rhythmic neural spiking by noise: the occurrence of a minimum in activity with increasing noise. Naturwissenschaften.

[CR15] Hodgkin AL, Huxley AF (1952). A quantitative description of membrane current and its application to conduction and excitation in nerve. J Physiol (Lond).

[CR16] Kim SY, Lim W (2015). Noise-induced burst and spike synchronizations in an inhibitory small-world network of sub-threshold bursting neurons. Cogn Neurodyn.

[CR17] Klasdin N (1995). Runge-Kutta algorithm for the numerical integration of stochastic differential equations. J Guid Control Dyn.

[CR18] Kuehn C (2015). Multiple time scale dynamics.

[CR19] Lindner B, Garcia-Ojalvo J, Neiman A, Schimansky-Geier L (2004). Effects of noise in excitable systems. Phys Rep.

[CR20] Longtin A (1993). Stochastic resonance in neuron models. J Stat Phys.

[CR21] Mahalanobis PC (1936). On the generalized distance in statistics. Proc Natl Inst Sci India.

[CR22] McLachlan G (2004). Discriminant analysis and statistical pattern recognition.

[CR23] Mil’shtein G, Ryashko L (1995). A first approximation of the quasi-potential in problems of the stability of systems with random non-degenerate perturbations. J Appl Math Mech.

[CR24] Paydarfar D, Forger DB, Clay JR (2006). Noisy inputs and the induction of on-off switching behavior in a neuronal pacemaker. J Neurophysiol.

[CR25] Pikovsky AS, Kurths J (1997). Coherence resonance in a noise-driven excitable system. Phys Rev Lett.

[CR26] Risken H (1989). The Fokker-Planck equation: methods of solution and applications.

[CR27] Tuckwell HC, Jost J (2009). Moment analysis of the Hodgkin-Huxley system with additive noise. Physica A.

[CR28] Tuckwell HC, Jost J, Gutkin BS (2009). Inhibition and modulation of rhythmic neuronal spiking by noise. Phys Rev E.

[CR29] Van Kampen NG (2007). Stochastic processes in physics and chemistry.

[CR30] Yamakou ME, Jost J (2018). Coherent neural oscillations induced by weak synaptic noise. Nonlinear Dyn.

